# A Novel Micronutrient Blend Mimics Calorie Restriction Transcriptomics in Multiple Tissues of Mice and Increases Lifespan and Mobility in *C. elegans*

**DOI:** 10.3390/nu12020486

**Published:** 2020-02-14

**Authors:** Eva Serna, Angela Mastaloudis, Patricia Martorell, Steven M. Wood, Shelly N. Hester, Mark Bartlett, Tomas A. Prolla, Jose Viña

**Affiliations:** 1 Freshage Research Group-Dept. Physiology-University of Valencia, CIBERFES, INCLIVA, 46010 Valencia, Spain; eva.serna@uv.es; 2 Pharmanex Research, NSE Products, Inc., Provo, UT 84601, USA; amastaloudis@nuskin.com (A.M.); stevew@nuskin.com (S.M.W.); shester@nuskin.com (S.N.H.); mrbartle@nuskin.com (M.B.); 3 Cell Biology Laboratory/ADM Nutrition/Biopolis SL/Archer Daniels Midland, 46980 Paterna, Valencia, Spain; Patricia.Martorell@adm.com; 4 LifeGen Technologies LLC, Madison, WI 53719, USA; taprolla@facstaff.wisc.edu; 5 Departments of Genetics and Medical Genetics; University of Wisconsin; Madison, WI 53706, USA

**Keywords:** aging, transcriptome, longevity, nutrition, micronutrient

## Abstract

Background: We previously described a novel micronutrient blend that behaves like a putative calorie restriction mimetic. The aim of this paper was to analyze the beneficial effects of our micronutrient blend in mice and *C. elegans,* and compare them with calorie restriction. Methods: Whole transcriptomic analysis was performed in the brain cortex, skeletal muscle and heart in three groups of mice: old controls (30 months), old + calorie restriction and old + novel micronutrient blend. Longevity and vitality were tested in *C. elegans*. Results: The micronutrient blend elicited transcriptomic changes in a manner similar to those in the calorie-restricted group and different from those in the control group. Subgroup analysis revealed that nuclear hormone receptor, proteasome complex and angiotensinogen genes, all of which are known to be directly related to aging, were the most affected. Furthermore, a functional analysis in *C. elegans* was used. We found that feeding *C. elegans* the micronutrient blend increased longevity as well as vitality. Conclusions: We describe a micronutrient supplement that causes similar changes (transcriptomic and promoting longevity and vitality) as a calorie restriction in mice and *C. elegans*, respectively, but further studies are required to confirm these effects in humans.

## 1. Introduction

Calorie restriction (CR), the reduction in calories typically by 40–60%, is a major intervention consistently demonstrated to retard aging and delay age-associated diseases [1,2]. However, when applied to humans, CR has serious shortcomings, including cold intolerance, weakness, impaired libido and a permanent feeling of hunger [3]. Furthermore, it is well established that it is difficult for humans to restrict calories or energy for extended periods of time. Therefore, recently there has been an increased focus on identifying CR mimetics, i.e., interventions including drugs, phytochemicals, vitamins, etc., that deliver the favorable effects of CR but without restricting macronutrient intakes, therefore avoiding the above-mentioned drawbacks. It is believed that CR confers benefits on healthspan and longevity via metabolic reprogramming [4]. Therefore, much of the focus in identifying CR mimetics has been on drugs that target metabolism and metabolic pathways. For example, drugs such as L-deoxyglucose that inhibit glycolysis and those that potentiate insulin action, such as metformin, have been investigated for their CR mimetic effects with varying success. However, the proposition of long-term treatments with drugs to promote lifespan, or even healthspan, may be controversial. Indeed, there may be some side effects associated with virtually any active drug that may prevent the physician from proposing such drugs as interventions in clinics with an endpoint of prolonging life. Instead, recent focus has been shifted to the identification of natural compounds—such as the phytochemical resveratrol [5]—that have a robust safety profile, but also exhibit some CR mimetic-like effects. However, to date, no single ingredient has been identified that can consistently deliver CR mimetic effects at the systemic level. Recently, our research group developed a screening tool utilizing transcriptional markers of calorie restriction “CR Supermarkers” to screen putative CR mimetic ingredients [6]. Using this method, we screened over 50 natural compounds for the ability to mimic CR at the transcriptional level in diverse tissues. Based on those results, we developed a micronutrient blend (see Table 1) to act as a putative CR mimetic. Our strategy in creating the blend of ingredients was to identify the vitamins and phytonutrients which mimicked CR at the transcriptional level most closely in individual tissues and then to combine those ingredients to form the final formula. For example, we observed in our screening that resveratrol showed very little CR mimicry in the muscle as compared to other ingredients like quercetin, yet demonstrated very high CR mimicry in the heart tissue of the mice.

Based on the results of the transcriptomic screening study, we identified those ingredients that demonstrated the highest CR mimicry for each tissue analyzed with the idea that such a blend would have the highest level of success as a CR mimetic at the systemic level. The aim of the present study was to test the ability of the micronutrient blend to mimic CR at the transcriptional level in mice as well as at the functional level in *C. elegans*. Here, we report the results of a transcriptomic analysis of brain, heart and skeletal muscle of old (30 months) controls, old treated with our micronutrient supplement and old calorie-restricted mice. We find that the transcriptome of calorie-restricted mice in all tissues studied is significantly different from that of old untreated ones. Remarkably, animals treated with the micronutrient blend show a transcriptomic profile that is similar to that of calorie-restricted animals, and significantly different from that of old, untreated animals. Furthermore, functional studies in *C. elegans* fed a diet supplemented with the micronutrient mixture confirmed both an increase in lifespan and improvements in vitality (locomotion) throughout different life stages.

## 2. Materials and Methods

### 2.1. Mouse Study Design

Three groups of B6C3F1 strain male mice; *n* = 7 per group, were studied; Old Controls (C) (fed AIN93M diet from 2–30 months of age), CR Group (CR) (fed AIN93M diet from 2–12 months of age, then fed a modified version of AIN93M diet with 25% energy restriction from 12–30 months of age), and Old Supplemented (S) (fed AIN93M diet from 2–12 months of age, then fed AIN93M diet plus a blend of natural ingredients from 12–30 months of age). Once the animals had reached a stable body weight at (12 months of age), animals were weighed monthly and diets adjusted accordingly to ensure that the mice maintained a stable body weight over the course of the study—with the exception of the CR group, that was allowed to lose weight beginning at 12 months of age.

All procedures were approved by the Institutional Animal Care and Use Committee at the William S. Middleton Memorial Veteran’s Hospital (Madison, WI, USA). The animals were euthanized by CO_2_ inhalation.

### 2.2. Micronutrient Blend

The composition of the micronutrient blend has been previously published [7] the blend is composed of: ultra-pure fish oil concentrate standardized to eicosapentaenoic acid (EPA) and decosahexanoic acid (DHA), resveratrol (from *Polygonum cuspidatum* root), quercetin (from *Dimorphandra mollis* fruit extract), purple corn (*Zea mays* L.) cob extract, rosemary (*Rosmarinus officinalis* L.) leaf extract, citrus bioflavonoids (naringin and hesperidin), coenzyme Q_10_, alpha lipoic acid, astaxanthin (a carotenoid from *Haematococcus pluvialis* algae), lycopene (a carotenoid), lutein (a carotenoid from Marigold flower (*Targetes erecta*), vitamin D_3_ (as cholecalciferol), vitamin K_2_ (as menaquinone-7) and d-limonene (from *citrus sinensis* peel oil); doses are described in Table 1.

### 2.3. Transcriptomic Study

Total RNA was extracted from brain cortex, skeletal muscle and heart using Trizol; the quality and integrity of total RNA was assessed by using the Bioanalyzer 2100 (Agilent Technologies, Inc., Santa Clara, CA, USA); all samples had an RNA integrity number (RIN) > 6. The GeneChip Mouse Gene 2.0 ST Array containing over 698,000 total probes constituting over 33,000 gene-level probe sets (>26,500 RefSeq genes) (Affymetrix, Santa Clara, CA, USA) was used for microarray analysis; one array per mouse per group for a total of 21 gene arrays. GeneChip^®^ Command Console^®^ Software (AGCC) supplied by Affymetrix was used to perform gene expression analysis.

### 2.4. Data Analysis of Microarrays

Data (CEL files) were analyzed and statistically filtered using Partek Genomic Suite 6.6 software (Partek Inc, St Louis, MO, USA). Data were normalized with the robust multiple array average (RMA) algorithm for background correction. Principal component analysis (PCA) was used to determine the significant sources of variability in the datasets. PCA reduces the complexity of high-dimensional data and simplifies the task of identifying expression patterns and sources of variability in a large dataset in a tridimensional fashion. The distance between any pair of points is related to the similarity between the 2 samples in high-dimensional space (in this case, each variable corresponded to a one-dimensional space). Samples in the plot close to each other are similar in a large number of variables, whereas samples far apart differ in a large number of variables.

Significant differentially expressed genes between CR or S vs. C were identified using a model one-way ANOVA with Partek Genomics Suite. Significant genes derived from the ANOVA analyses were visualized according to their expression levels in an unsupervised hierarchical clustering.

Finally, the selected differentially expressed genes were imported into Pathway Studio software version 10 (Elsevier Inc., Rockville, MD, USA) to identify the main master regulators using subnetworks analysis with database resnet 11.

The microarrays are stored in ArrayExpress with accession number MTAB-8506. (https://www.ebi.ac.uk/arrayexpress/).

### 2.5. C. elegans Study

#### 2.5.1. Sample

The micronutrient blend was provided in liquid hard capsules, the content of which was used to prepare the stock solution in milliQ water at 10%, after the vortex agitation of the capsules. This solution was used to prepare the different doses of the micronutrient blend in the nematode culture plates (NGM). Thus, different volumes (5, 10, 30 and 100 µL were added on the agar surface to obtain doses of 0.005%, 0.01%, 0.03% and 0.1%, respectively. Moreover, in order to rule out any toxicity of the supplement on the in vivo model, different doses of the supplement were tested: 0.005%, 0.01%, 0.03% and 0.1%; viability, development of the different larval stages and progeny were checked from the egg to the young adult stage.

#### 2.5.2. Lifespan experiments in *C. elegans*

The wild-type strain of *C. elegans* (N2) was age-synchronized by recovering the eggs from adults in the corresponding agar plates already seeded with Escherichia coli OP50. Nematode Growth Medium (NGM) was used as control diet, and the supplement was added to the NGM medium at the different doses (0.005%; 0.01%; 0.03% and 0.1%). Nematodes (100/condition/assay) were incubated at 20 °C during 25 days in the different media, moving them to new plates every two days. Survival was scored during this period, and worms were scored as dead if they failed to respond to a platinum wire.

Survival curves of those conditions providing positive results were compared using the log rank survival significance test, provided by GraphPad Prism 4 statistical software package. Experiments were performed in duplicate.

#### 2.5.3. Locomotion Activity in *C. elegans*

The nematode locomotion is characterized by sinusoidal movement, and it is an age-related parameter. Different methods have been described to measure *C. elegans* locomotion. The most frequently used is the quantification of body bends in a specific period of time. Using this technique, a movement rate can be determined in a worm population under specific treatments.

Here, the effect of the micronutrient blend was analyzed in vivo to determine if the treated population displayed higher mobility (vitality) than the control fed group.

Experiments were carried out with *C. elegans* wild-type strain (N2). Age-synchronized worms were cultured in the corresponding media: Nematode Growth Media (NGM, control), and NGM supplemented with three doses of the micronutrient blend (0.01%, 0.03% and 0.1%).

Mobility was analyzed on 1-day- (young adult stage) and 3-day-old adults (old adult stage). The mobility rate was measured under dissecting scope by counting the number of body bends produced by individual worms in 40 s. The average of body bends and the mobility frequency in the population was analyzed by One-Way ANOVA (Tukey’s Multiple Comparison Test). Experiments were performed in duplicate or triplicate for each dose tested.

## 3. Results

### 3.1. Tridimensional Principal Component Analysis of Whole Genome Transcriptomics of CR and a Micronutrient Blend Formulation in Brain Cortex, Skeletal Muscle and Heart of Mice

**Figure 1:**
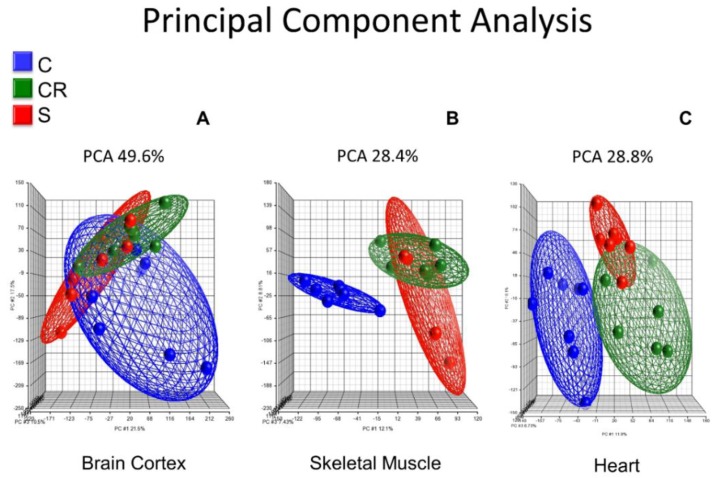
Principal component analysis of the whole transcriptome of old mice (C) (blue), old calorie restricted (CR) (green) and old supplemented with a calorie restriction mimetic micronutrient blend (S) (red) in brain cortex (Panel A), skeletal muscle (Panel B) and heart (Panel C). The percentage results of the sum of the three components (PC1, *x*-axis), PC2 (*y*-axis), and PC3 (*z*-axis) as indicated in the figure (% = PC1 + PC2 + PC3). The percentage of variability is 49.6% in brain cortex, 28.4% in skeletal muscle and 28.8% in heart.

Figure 1 shows the principal component analysis (PCA) of the whole transcriptome of brain cortex (Panel A), skeletal muscle (Panel B) and heart (Panel C) of old mice, old calorie-restricted mice and old mice treated with the micronutrient blend formulation (see Table 1).

Panel A shows the PCA in brain cortex where old untreated animals (in blue) cluster in a completely different manner than either calorie-restricted (in green) or old animals supplemented with the CR mimetic (in red). In contrast, the latter two cluster in a very similar way, and thus the red and green ellipsoids show a similar profile.

Skeletal muscle transcriptome of old animals clusters in a different manner when compared with the calorie-restricted or the ones treated with the CR mimetic: the blue ellipsoid is completely separated from the red or the green and the old animals supplemented with the CR mimetic cluster with the old CR group (Panel B).

In a similar fashion, Panel C shows the principal component analysis of the heart transcriptome, and as is the case with brain and skeletal muscle, heart muscle transcriptomic analysis shows that old animals, calorie-restricted or those fed with the CR mimetic cluster in a completely separate way than calorie-restricted and those fed with the CR mimetic. Notably, those in the CR and CR mimetic groups cluster together, consistent with the brain cortex and skeletal muscle results.

The major result that we show in Figure 1 is that the brain cortex, skeletal muscle and heart transcriptomic profiles of the old animals cluster in a distinct way to that of calorie-restricted and old treated with the CR mimetic, whereas the calorie restricted and micronutrient blend groups exhibit similar transcriptomic patterns.

### 3.2. Differential Analysis of Whole Genome Transcriptomic of CR and a Micronutrient Blend Formulation in Brain Cortex, Skeletal Muscle and Heart of Mice

**Figure 2:**
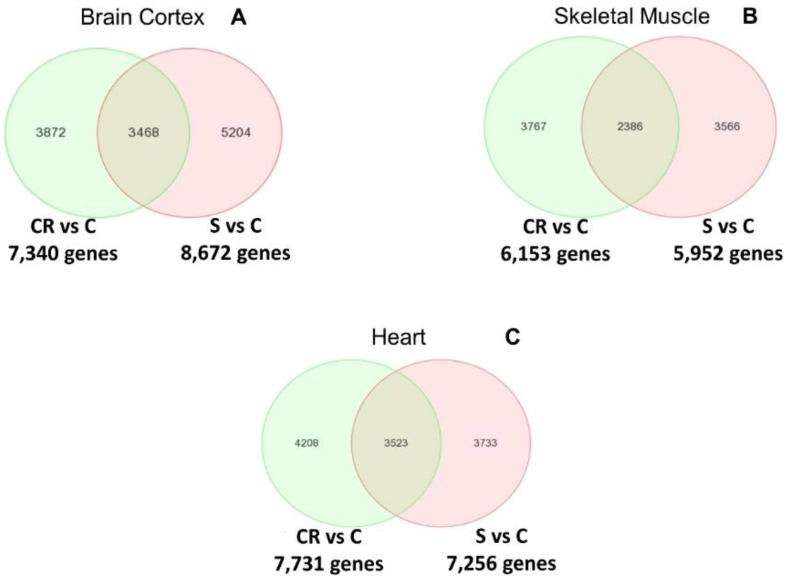
Venn diagrams of the differentially expressed genes that overlap between old calorie restricted (CR) vs. (C) and old supplemented with a calorie restriction mimetic (S) vs. old mice (C) in brain cortex (Panel A), skeletal muscle (Panel B) and heart (Panel C).

Figure 2 shows the Venn diagrams of the differential analysis of the whole transcriptome of old animals, old subjected to CR and old treated with the CR mimetic.

Panel A indicates the differentially expressed genes between both comparisons in brain cortex: CR vs. old controls (CR vs. C) and supplemented vs. old controls (S vs. C). The Venn diagram in panel A indicates that more than 3400 genes overlap. The complete list of genes can be consulted in the Table S1.

In the case of skeletal muscle, establishing the same comparisons as with the brain cortex, over 2300 genes are commonly regulated by CR or by feeding the supplemented diet to animals (see Panel B). The complete list of genes can be consulted in Table S2.

Finally, Panel C shows that in the case of the heart, over 3500 genes are commonly regulated in CR and in animals fed with the supplemented diet. The complete list of genes can be consulted in the Table S3.

We thus report in Figure 2 that CR and the CR mimetic micronutrient blend result in the common regulation of a very large number of genes in brain cortex, skeletal muscle and heart.

### 3.3. Unsupervised Hierarchical Clustering Analysis of Whole Genome Transcriptome of CR and a Micronutrient Blend Formulation in Brain Cortex, Skeletal Muscle and Heart of Mice

**Figure 3:**
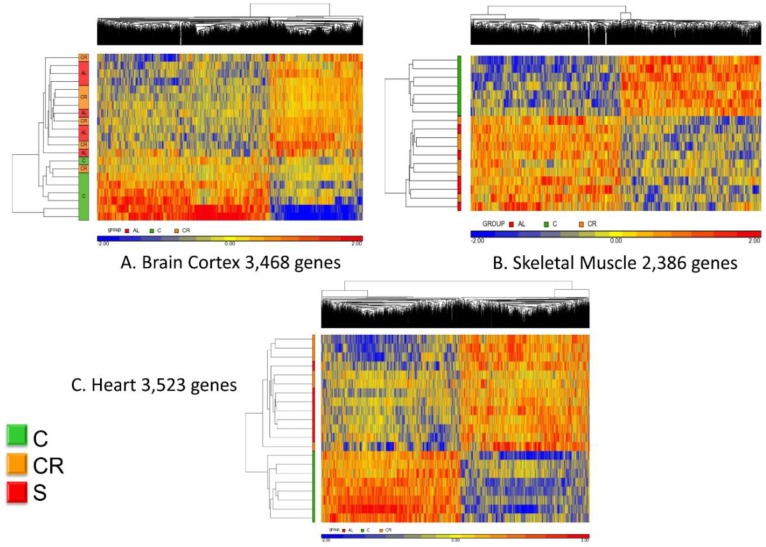
Unsupervised hierarchical clustering (heatmaps) of the differentially expressed genes of old mice (C), old calorie restricted (CR) and old supplemented with a calorie restriction mimetic (S) in brain cortex, skeletal muscle and heart. The number of the differentially expressed genes for each comparison is shown in Figure 2. Overexpressed genes are represented in red and underexpressed genes in blue. Each line represents a tissue sample and each column a gene.

Figure 3 shows the unsupervised hierarchical clustering analysis of the whole transcriptome.

Panel A refers to the brain cortex where the 3468 genes that were commonly regulated (see Figure 2) are now analyzed in terms of their hierarchical clustering for each individual animal. With one exception, all old controls clustered together whereas the calorie-restricted and animals fed with the CR mimetic are interspersed and indistinguishable.

The same analysis was performed using the 2386 genes that were commonly expressed in skeletal muscle and a similar pattern emerged: all of the controls clustered together (represented in this figure in green in the left vertical bar) whereas the CR and CR mimetic animals were interspersed and were nearly indistinguishable from one another.

Finally, analysis of the heart transcriptomic revealed a similar consistent pattern as brain cortex and skeletal muscle. Based on the 3523 genes identified by the differential analysis, the CR animals and those treated with the CR mimetic are interspersed and the controls are clearly different.

Based on these results, we conclude that the micronutrient blend formulation appears to be a CR mimetic, as it elicited a transcriptomic profile nearly indistinguishable from that of CR group when compared with old, untreated control animals.

### 3.4. Subnetwork Analysis of the Brain Cortex, Skeletal Muscle and Heart Genes that Were Differentially Expressed in Both CR and in a Micronutrient Blend Formulation-Fed Group When Compared with Control Animals

**Figure 4:**
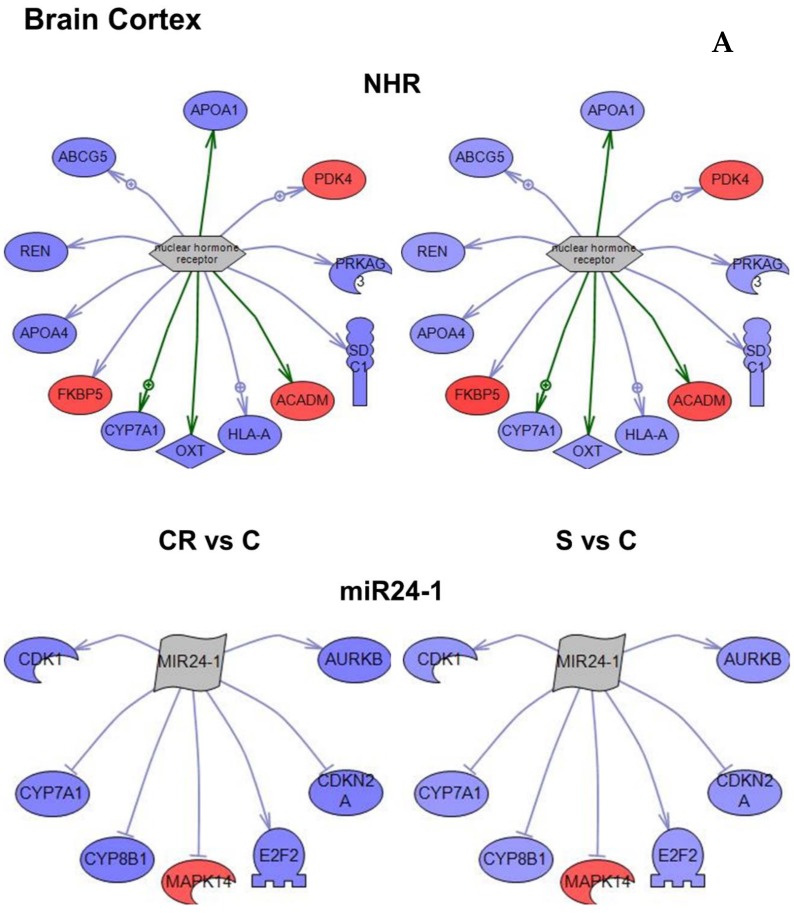

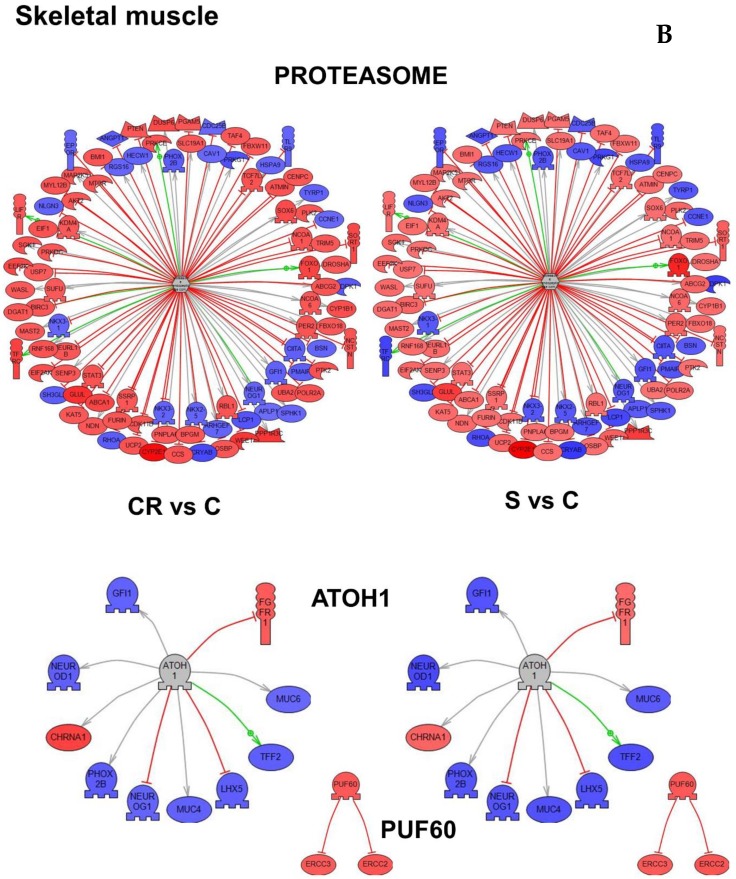

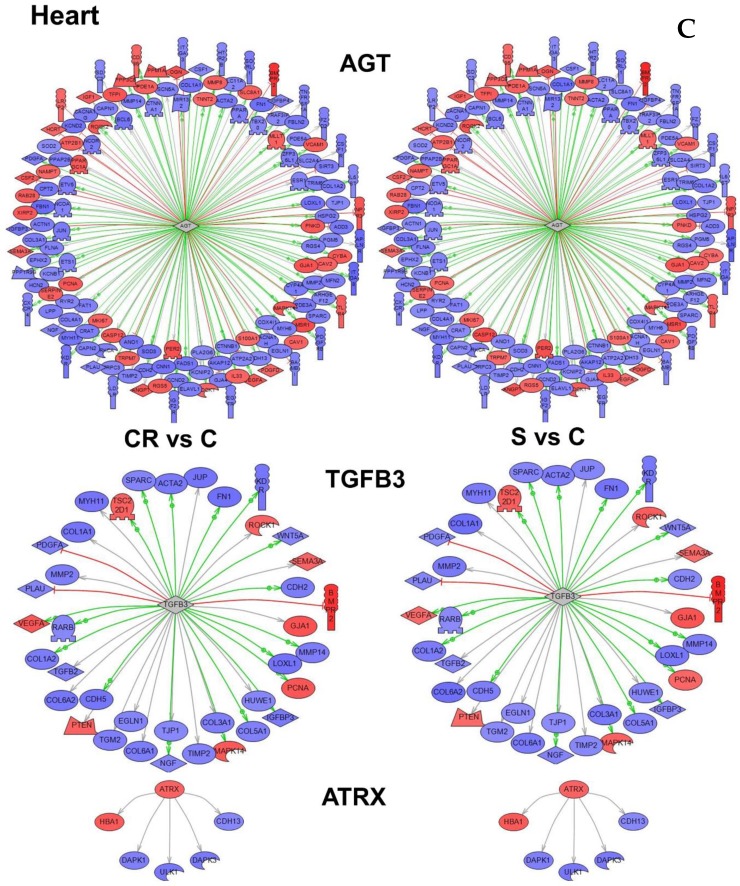
(**A**) Subnetworks enrichment analysis for upstream regulators of expression classified for overlap or *p*-value in brain cortex. The regulators are shown as nodes with downstream interactions indicated by edges. Colors correspond to a level of expression (blue is underexpression and red is overexpression). Color of the edges corresponds to a type of regulation of expression. Green is known positive regulation, red is known negative regulation and grey is a direct regulation, but unknown effect. Representative subnetworks of the overlap or *p*-value are shown. (**B**) Subnetworks enrichment analysis for upstream regulators of expression classified for overlap or *p*-value in skeletal muscle. The regulators are shown as nodes with downstream interactions indicated by edges. Colors correspond to a level of expression (blue is underexpression and red is overexpression). The color of the edges corresponds to a type of regulation of expression. Green is known positive regulation, red is known negative regulation and grey is a direct regulation, but unknown effect. Representative subnetworks of the overlap or *p*-value are shown. (**C**) Subnetworks enrichment analysis for upstream regulators of expression classified for overlap or *p*-value in heart. The regulators are shown as nodes with downstream interactions indicated by edges. Colors correspond to a level of expression (blue is underexpression and red is overexpression). Color of the edges corresponds to a type of regulation of expression. Green is known positive regulation, red is known negative regulation and grey is a direct regulation, but unknown effect. Representative subnetworks of the overlap or *p*-value are shown.

A “subnetwork” is a group of genes connected structurally or functionally to one common gene based on known relationships between the genes. Using the genes identified with the differential analysis (Figure 2) several subnetworks were identified in each tissue analyzed using the Pathway Studio^®^ database (Elsevier Inc., Rockville, MD, USA) (Tables S4–S6). The list of genes in terms of overlap (i.e., genes that are all related to the same gene), and the number of genes in terms of enrichment p value are represented in the Supplementary Materials (Tables S4–S6) and were subsequently used to identify the most robustly changed subnetwork within each tissue (Figure 4A–C).

Based on the subnetwork analysis of the 3468 genes that were differentially regulated in CR or in animals supplemented with the CR mimetic when compared with old controls, the nuclear hormone receptor and MIR24-1 were identified as the most robust subnetworks in brain cortex (Figure 4A and Table 2).The subnetwork that had the greatest number of genes and the one with the highest *p* value were each selected (Table S4) since both approaches contribute to our understanding of the changes caused by either CR or the treatment with the CR mimetic.

Figure 4B represents the subnetworks for two genes in skeletal muscle, PUF60 (Poly(U)-binding-splicing factor) and ATOH1 (Protein atonal homolog 1) which were selected based on p-value as well as the proteasome subnetwork, the functional class of genes demonstrating the highest overlap (Table S5).

The relevant subnetworks identified by the subnetwork analysis for the 3523 genes differentially expressed in the heart are shown in the Supplementary Materials (Table S6). Figure 4C depicts the subnetworks for three genes. The first, angiotensinogen (AGT), had highest overlay in number and highest *p* value (Table S6)—a strong indication that angiotensinogen is an important gene in the functional regulation of the heart tissue. The second and third genes—ATRX (ATP-dependent helicase ATRX) and TGFB3 (Transforming growth factor beta-3)—were both selected based on higher *p* values.

An important common feature can be observed in Figure 4 that genes within each subnetwork tend to change in the same direction in both CR and in animals supplemented with the CR mimetic (in blue underexpression and in red overexpression). The Tables S4–S6 show genes involved in each subnetwork, and the level of expression can be consulted in Tables S1–S3.

The conclusion is that the transcriptomic profile of animals subjected to CR is similar to that of old animals treated with our CR mimetic.

In Table 2, we summarize all subnetworks analyses. We represent the tissue in which we have identified a given gene as a “master regulator of a subnetwork” (see Figure 4), the relevant biological processes and comments refer to the relation of a gene or functional class of genes with aging.

**Figure 5:**
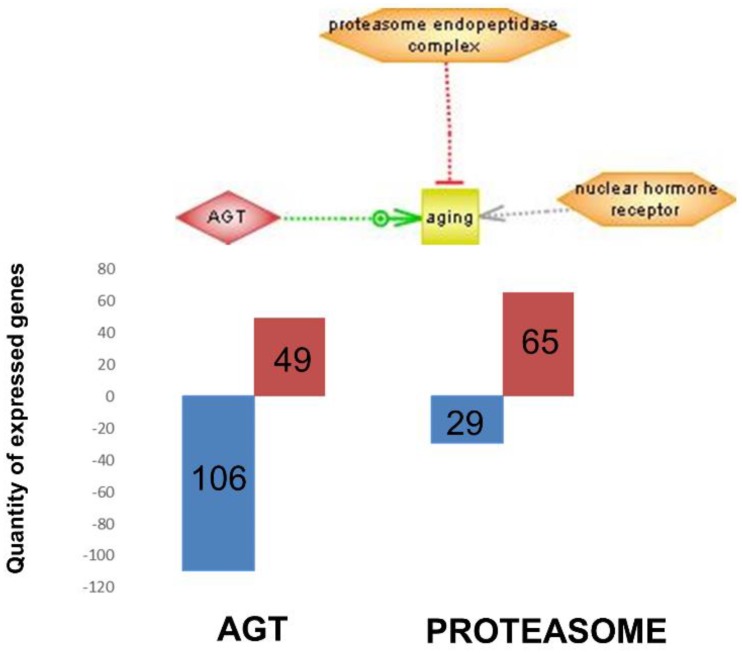
Representation of some of the most important regulators found (see Figure 4) that are related to aging and the total number of genes that are down or overexpressed in this subnetwork. Color of the arrow corresponds to the type of regulation of expression. Green is known positive regulation, red is known negative regulation and grey is a direct regulation, but unknown effect. Bar columns in blue are genes underexpressed and bar columns in red are genes overexpressed for angiotensinogen (AGT) and proteasome subnetworks.

Figure 5 integrates the most important regulators found (nuclear hormone receptor identified in brain cortex; proteasome endopeptidase complex, identified in skeletal muscle and angiotensinogen, identified in heart, see Figure 4 respectively).

### 3.5. Effect of the Micronutrient Blend on C. elegans Lifespan

In a first step, the toxicity of the supplement was examined at different doses of the micronutrient blend (0.005%, 0.01%; 0.03%; 0.1%) on *C. elegans* reproduction and development. No adverse effects were observed with the micronutrient blend, and only a slight, non-statistically significant, reduction in progeny was observed at the highest dose (1%) (data not shown). Consequently, the efficacy of the micronutrient blend on lifespan was determined at the various doses of 0.005%, 0.01%; 0.03%; and 0.1% (wt. of supplement per volume of the *C. elegans* diet). No effects were observed at the lowest dose tested i.e., 0.005% for any parameter; therefore, these data are not shown.

**Figure 6:**
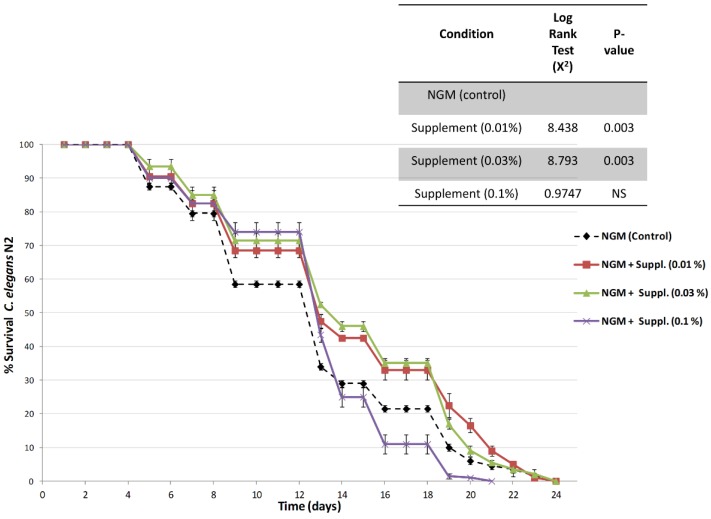
Survival curves (percentage of survival) obtained in *C. elegans* (N2) cultured in nematode culture plates (NGM) or in NGM with different doses of the supplement. Data are the average of two independent experiments.

Figure 6 shows the survival curves, an indication of lifespan, obtained in nematodes cultured in nematode growth media (NGM, control) and in NGM supplemented with different doses of the micronutrient blend formula.

Results indicate an increase in the lifespan of nematodes treated with the micronutrient blend formula at both 0.01% and 0.03% doses. Furthermore, log rank testing indicated that lifespan of animals fed the micronutrient blend formula at doses of 0.01% or 0.03% lived significantly longer than controls or nematodes fed with the highest dose of the micronutrient blend. Thus, when compared the overall survival curves against control-fed nematodes, the survival was highly significant (*p* = 0.003), and this increase in survival was observed during the initial, medium and final lifespan (Table 3). The nematodes fed the highest dose, 0.1%, exhibited a survival curve similar to that of the controls.

### 3.6. Effect of the Supplement on C. elegans Locomotion

**Figure 7:**
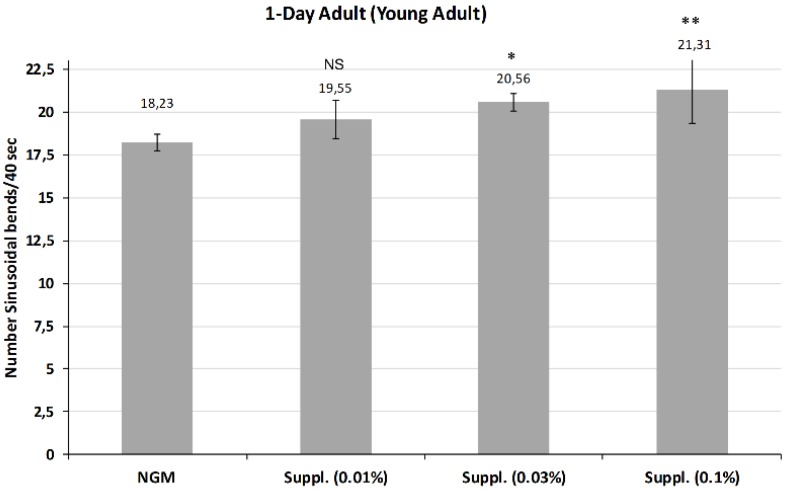
Mobility rate of *C. elegans* N2 young adults (1-Day) treated with the supplement at different doses. Number of body bends in 40 s measured in a total of 25 worms/condition/assay. ** Significant at *p* ≤ 0.01; * Significant at *p* ≤ 0.05; NS: Not significant vs. control NGM. Data are the average of two or three independent experiments.

**Figure 8:**
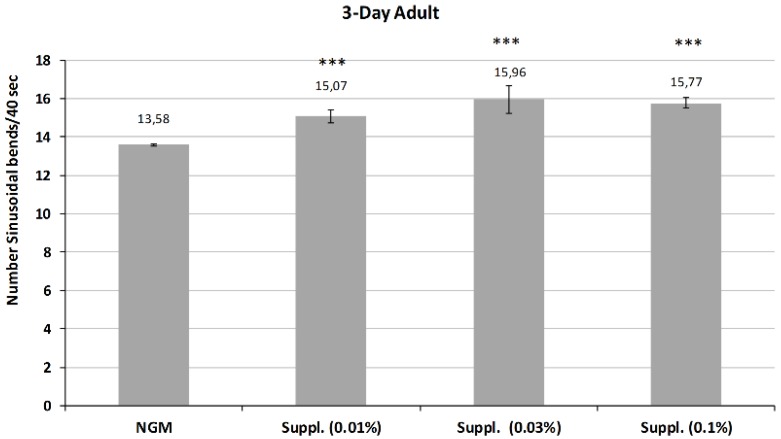
Mobility rate of *C. elegans* (N2) at 3-Day adult stage treated with the supplement at different doses. Number of body bends during 40 s measured in a total of 25 worms/condition/assay. *** Significant at *p* ≤ 0.001 vs. control NGM. Data are the average of two or three independent experiments.

In *C. elegans*, the well-coordinated sinusoidal body movement characteristic of young hermaphrodites typically becomes progressively slower and less coordinated with age, as early as day one of life. In our study, we determined locomotion, an indication of vitality, at day 1 of life (Figure 7) and at day 3 (Figure 8) of life. The effect of the supplement at the doses of 0.01, 0.03 and 0.1%, was studied.

Figure 7 shows the average of body bends measured in young adults after treatment with different doses of the micronutrient blend. Results indicated that higher doses of 0.03% and 0.1% both provided a significant increase in mobility compared with controls at day 1 of life. Notably, the dose of 0.1% was the most effective, increasing the mobility by 16.8% (*p* < 0.01), although this dose had no effect on lifespan. No effect was observed at the 0.01% dose.

Subsequently, the activity of the micronutrient blend was studied in the 3-Day adults, whose mobility is reduced in control conditions due to aging.

The results, shown in Figure 8, indicate the maintenance of or even an increase in the revitalizing effect of the micronutrient blend at this stage of life. Thus, all the doses of micronutrient blend provided a significant increase in body bends on locomotion, a biomarker of vitality (*p* < 0.001) compared with control conditions.

### 3.7. Effect of the Micronutrient Blend on C. elegans Bends Frequency

Due to the potential intra-population variability in the mobility of the worms, bends frequency was studied under the different conditions. Different groups were established, taking into account the number of body bends in 40 s (an indicator of speed of worms and another biomarker of vitality). Thus, worms with ≥25 body bends/40 s were the fastest, and those with ≤10 bends/40 s the slowest.

**Figure 9:**
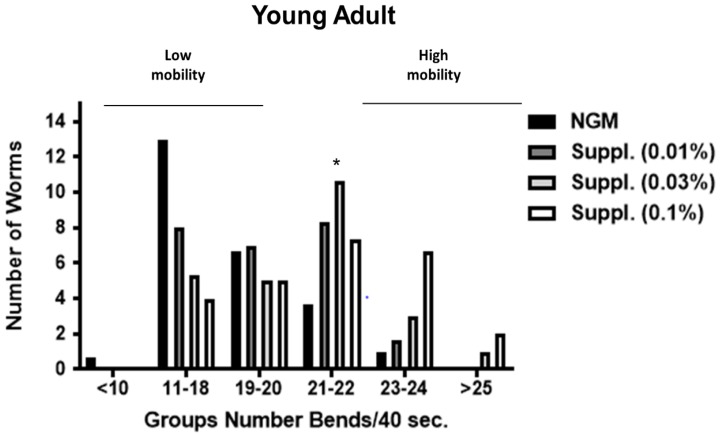
Distribution of mobility rate in Young adult (1-Day) worms used in the study. Different groups were established based on the speed (number of body bends/40 s) of worms. One-way ANOVA was used to compare conditions within each group for number of body bends. * Significant *p* < 0.05 vs. NGM (within the group).

**Figure 10:**
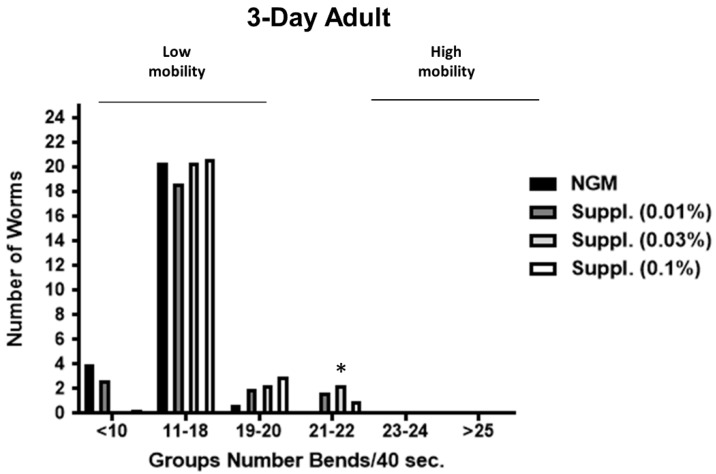
Distribution of mobility rate in 3-Day Adult worms used in the study. Different groups were established based on the speed (number of body bends/40 s) of worms). One-way ANOVA was used to compare conditions within each group for the number of body bends. * Significant *p* < 0.05 vs. NGM (within the group).

In this analysis, our results indicate that supplementation promotes a clear increase in the abundance of worms with high mobility (23–24 or >25 bends/40 s), especially at the dose of 0.1% (Figure 9 and Figure 10). Thus, in the group with 23–24 bends/40 s, those fed a dose of 0.1% of supplement contained significantly greater numbers of ‘fast’ worms compared to the control group (*p* < 0.05) (Figure 10). This was more evident in young adult stages (1-day adult) than in the 3-Day adults, probably due to the natural drop of locomotion at 3 days into the experiment (Figure 9 and Figure 10). Thus, in 1-day old worms, those fed a dose of 0.1% of supplement contained significantly greater numbers of ‘fast worms (the group with 23–24 bends/40 s) compared to the control group (*p* < 0.05) whereas the dose of 0.03% increased the number of worms moving at 21–22 bends/40 s) (*p* < 0.05) (Figure 9).

In the case of the 3-Day adults (Figure 10), the supplement at a dose of 0.1% again led to an increase in the number of worms with high mobility (group of 19–20 bends/40 s) and the dose of 0.03% increased the number of worms moving at 21–22 bends/40 s) (*p* < 0.05).

Overall, the micronutrient blend investigated in this study led to positive effects on the longevity and vitality, locomotion and speed, of *C. elegans* (Table 4, Figure 6). Among the three doses included in the experimental design (0.01%, 0.03% and 0.1%), the doses of 0.03% provided robust and significant effects on longevity and increased vitality. Moreover, this dose also led to improvements in mobility parameters that were maintained from young adulthood into adult stages characterized by a decline of health parameters, including speed and coordination. In contrast, the 0.1% dose, improved vitality above and beyond controls as early as day 1, an effect that persisted at 3 days and that translated to superior speed. However, despite this positive effect on vitality, the highest dose did not impact longevity, probably due to the chronic exposition of the treatment during lifespan assays. In fact, the treatment resulted in innocuous (and positive) changes during the first middle of lifespan (see Table 4, Figure 6).

## 4. Discussion

CR is one of the most consistent and powerful interventions to retard aging and prolong lifespan, at least in experimental animals, including non-human primates. This phenomenon was described by McCay in the 1930s [1] and has been thoroughly investigated during the majority of the 20th and 21st centuries [2]. However, the molecular mechanisms underlying the favorable effects of CR to promote longevity and health are poorly understood. Moreover, CR implies lowering intake of calories but without malnutrition, requiring fortification or supplementation of the diet with essential nutrients. Another major issue is that it is very difficult for humans to follow a caloric restricted diet due to the drawbacks such as feeling uneasy, lowering of sexual impetus, feeling cold, constant hunger, etc. Therefore, CR can be used as a model to understand healthy aging, but CR mimetics would be appealing, i.e., interventions that elicit positive cause effects that are similar to CR but without the drawbacks. The term CR mimetic was first used by Lane, Ingram and Roth of the National Institute on Aging in 1998 [21]. In general, CR mimetic studies have focused on individual substances like metformin [22] or SRT1720, a SIRT-1 Activator [23]. For a recent review on this topic, see Madeo et al. 2019 [24].

In the present study, we report that a mixture of micronutrients, as far as the transcriptomic signature in mice and lifespan and mobility in worms are concerned, mimics the effect of CR [4,25]. Our group first described the gene expression profiles of aging and the effect of CR. These results have been validated by several laboratories and show that transcriptomic analysis is a useful tool to evaluate the role of interventions to retard the effects of aging and to understand the biological processes involved. The ingredients in the micronutrient blend investigated in the present study were selected using a screening method developed by our group designed to identify putative CR mimetics utilizing transcriptional markers of CR “CR Supermarkers” [6]. Using this method, we screened over 50 natural compounds for the ability to mimic CR at the transcriptional level in diverse tissues, of these, 15 ingredients were selected for inclusion in the micronutrient blend studied herein.

We report here results for brain cortex, heart and skeletal muscle, three tissues known to be heavily influenced by the aging process. The general conclusion that can be drawn from this mouse study is that the micronutrient blend that we show in Table 1 changes the transcriptome with aging in these three organs, most relevant to aging, in a way that is very similar to that caused by CR when compared with the transcriptomic profile of untreated old mice.

An important point to highlight is that the effects are systemic in that they are not limited to one organ, but impacted all the organs studied—brain, skeletal muscle and heart. It must be stressed that the biological orientation of CR and of feeding the animals with the CR mimetic is similar, i.e., genes that are overexpressed in CR are also overexpressed in CR mimetic-fed animals.

Furthermore, the subnetwork analysis led us to identify three major master regulator genes (i.e., genes that are functionally or structurally related to others differentially expressed with the intervention, in this case dietary supplementation). Figure 5 shows these three genes (nuclear hormone receptor identified in brain cortex; proteasome endopeptidase complex, identified in skeletal muscle and angiotensinogen, identified in heart, see Figure 4 respectively). It is noteworthy that angiotensinogen (that is directly associated with aging) is downregulated in the heart of supplemented animals, whereas proteasome (that is inversely associated with aging) is upregulated in heart of supplemented animals.

Furthermore, the subnetwork analysis led us to identify three major master regulator genes (i.e., genes that are functionally or structurally related to others and they are also differentially expressed with the intervention, in this case dietary supplementation). Figure 5 shows these three genes (nuclear hormone receptor identified in brain cortex; proteasome endopeptidase complex identified in skeletal muscle; angiotensinogen identified in heart, see Figure 4, respectively). It is noteworthy that angiotensinogen (that is directly associated with aging) is downregulated in the heart of supplemented animals whereas proteasome (that is inversely associated with aging) is upregulated in heart of supplemented animals.

These results provide evidence that nutritional supplementation, in the form of a micronutrient blend, may promote healthy aging at least in terms of gene expression. In addition, these results suggest that our screening tool [6], based on CR transcriptomics, effectively identified ingredients with CR mimetic effects.

The favorable effects of our micronutrient blend on the transcriptomic profile prompted us to test its functional effects on longevity and vitality (speed and locomotion) using *C. elegans*, a convenient and well-established experimental model. *C. elegans* is a popular model in aging research because it shares many of its genes with humans and because its short lifespan of only 21 days, allowing scientists to quickly assess the effects of genetic and environmental interventions to extend healthy lifespan [26]. Moreover, parameters like mobility and locomotion are frequently used in the *C. elegans* community to assess healthy status (even to rule out toxic effects) [27]. Nematodes’ mobility is a parameter related with neuronal function, and therefore very relevant for cognitive aging [28].

We used three different doses of the micronutrient blend. Figure 6 and Table 3; Table 4 show that the percent increase in longevity—which is very significant (up to 15%)—already occurs at low doses of the supplement (0.01%), is maximal at 0.03% and diminishes at the higher dose (0.1%).

The functional effects observed in the nematode, indicate that further experiments are required to test if these effects also occur in mammalian species, i.e., mice and humans.

Although some studies have reported the CR mimetic effects of resveratrol and other individual micronutrients included in the blend, the results have been inconsistent, with some studies reporting healthspan benefits, others lifespan benefits, and others no benefit [29,30,31,32,33]. Thus, the CR mimetic effects of individual micronutrients included in the blend are not definitive. Furthermore, the majority of studies of resveratrol and other ingredients included were previously studied at much higher doses [6]. For example, the majority of clinical studies demonstrating beneficial effects of resveratrol were previously studied in the 150–5000 mg/day range [34], compared to only 30 mg human equivalent dose in the present study. Moreover, the resveratrol is only a minor component of the overall blend. If one to were calculate the amount delivered in the growth media of the *C. elegans* at the different doses (0.01%, 0.03% and 0.1%) the amount of resveratrol is (0.000092%, 0.00028% and 0.00092%) which is lower than previous studies [35]. Additionally, clinical studies showing health benefits for astaxanthin are typically 2–8 mg/day [36] compared to the 1 mg/day human equivalent dose in current study, and clinical studies demonstrating health benefits for quercetin are typically 100–730 mg/day [37] compared to 75 mg/day human equivalent dose in current study. There is no evidence that these low concentrations of only resveratrol or other ingredients in the blend would increase longevity, healthspan or vitality in *C. elegans* or other model systems. The observation that the blend included much lower levels of the individual ingredients yet delivered positive effects in humans, in situ in mice, and *C. elegans*, suggests a synergistic effect of the combined ingredients rather than the result of any single ingredient. However, some pathways observed to be impacted in this study are pathways that have been previously characterized to be affected by resveratrol and other micronutrients included in the blend (see Table S7). However, if resveratrol was the only active ingredient in the blend, then sirtuins [38] would have likely been identified in our analysis as critical genes, which they were not.

The present studies also provide evidence for the safety of the micronutrient blend. The mice were fed the micronutrient blend for 18 months with no differences in mortality or morbidity between any of the groups, controls, calorie restricted or supplemented by the time of sacrifice, 30 months (data not shown). In addition, in establishing the doses for the *C. elegans* study, no toxic levels of the supplement were identified (see methods). Furthermore, the safety as well as the efficacy of this mixture for other biomarkers of health has been tested and confirmed in humans [7], but experiments investigating the possibility that this CR mimetic may be useful to increase the healthspan long-term in humans remains a longer-term goal.

The major finding reported is that we developed a novel micronutrient blend that induces transcriptomic changes in mice that are similar to those induced by calorie restriction. Moreover, when this blend is fed to *C. elegans*, it increases both the lifespan and vitality of the worms. Further studies to test if this is also the case in mice are going to be carried out in our laboratories.

## Figures and Tables

**Table 1:** Micronutrient blend formulation.

Components	Human Equivalent Dose (Reagan Shaw) (amount per day)	Amount in Mouse Diet (mg/kg bw)
Fish Oil Concentrate (1000 mg EPA + DHA)	2110 mg	372
Vitamin K_2_ (menaquinone-7)	40 mcg	0.007
Vitamin D_3_	25 mcg (1000 IU)	0.0044
Lycopene	5 mg	0.88
Lutein	4 mg	0.70
Astaxanthin	1 mg	0.18
Hesperidin, Citrus Bioflavanoid	100 mg	17.6
Naringin, Citrus Bioflavanoid	100 mg	17.6
Purple Corn Extract (std. to anthocyanins)	133 mg	26
Alpha Lipoic Acid	100 mg	17.6
Quercetin	75 mg	13.2
d-Limonene	50 mg	8.8
Rosemary Leaf Extract (std. carnosic acid)	37.5 mg	6.6
Resveratrol	30 mg	5.3
Coenzyme Q_10_	30 mg	5.3

**Table 2:** Function of master regulator genes identified in the tissues studied.

Gene/Functional Class	Target Tissue	Biological Process	Comments
Nuclear hormone receptor	Brain Cortex	Regulation of transcription. Control the development and differentiation of skin, bone and behavioral centers in the brain, and the regulation of reproductive tissues.	Related to aging but mechanisms unknown.
miR-24-1 (aka miR-189)	Brain Cortex	Regulation of transcription. Regulation of apoptosis	A miRNA-24 inhibitor prevents apoptosis in ischemic stroke by regulation of Bcl-xL, Caspase-3 and HSP70 [8].
Proteasome endopeptidase complex	Skeletal muscle	Degradation of cytosolic and nuclear proteins in eukaryotic cells.	Negative regulation aging [9,10,11,12,13].
PUF60 (Poly(U) binding splicing factor 60) (aka FIR)	Skeletal muscle	RNA splicing Regulation of transcription Apoptotic process	
ATOH1 (Atonal bHLH transcription factor 1)	Skeletal muscle	Regulation of transcription (transcription factor) Apoptotic process Cell differentiation	Inhibits proteasome endopeptidase complex [14].
AGT (Angiotensinogen) (aka Serpina8)	Heart	Aging Regulation of blood pressure Cytokine secretion Cardiac muscle cell development	Activates aging process [15,16,17,18].
ATRX	Heart	DNA methylation DNA repair	AGT positive regulation [19].
TGFB3 (Transforming growth factor, beta 3)	Heart	Blood coagulation Response to hypoxia Cell growth Regulation of apoptotic process	AGT positive expression [20].

**Table 3:** Effect of supplement on the initial, mean and final lifespan.

Condition	Initial Lifespan (Day 65% Survival)	Mean Lifespan (Day 50% Survival)	Final Lifespan (Day 25% Survival)
**NGM (control)**	8.8	12.3	15.9
**Suppl. 0.01%**	12.4	13.0	19.0
**Suppl. 0.03%**	12.4	13.5	18.7
**Suppl. 0.1%**	12.4	13.0	14.0

**Table 4:** Summary of the functional activity provided by the micronutrient supplement to *C. elegans*.

Micronutrient Blend Dose (%)	Increased Longevity (Day 15)	Increased Mobility (1-Day Adults)	Increased Mobility (3-Day Adults)
**0.01**	13% (*p* < 0.003)	7% (NS)	10.9% (*p* ≤ 0.001)
**0.03**	15% (*p* < 0.003)	12.7% (*p* ≤ 0.05)	17.5% (*p* ≤ 0.001)
**0.1**	NS	16.8% (*p* ≤ 0.01)	16.1% (*p* ≤ 0.001)

## Supplementary Materials

The following are available online at https://www.mdpi.com/2072-6643/12/2/486/s1, Table S1: Differentially expressed genes between CR vs. C and S vs. C in brain cortex. Table S2: Differentially expressed genes between CR vs. C and S vs. C in skeletal muscle. Table S3: Differentially expressed genes between CR vs. C and S vs. C in heart. Table S4: The relevant subnetworks filtered for enrichment p-value and overlap in brain cortex. Table S5: The relevant subnetworks filtered for enrichment p-value and overlap in skeletal muscle. Table S6: The relevant subnetworks filtered for enrichment p-value and overlap in heart. Table S7: The relevant pathways filtered for enrichment p-value in brain cortex, skeletal muscle and heart.
