# Supplementary material for: A Novel Micronutrient Blend Mimics Calorie Restriction Transcriptomics in Multiple Tissues of Mice and Increases Lifespan and Mobility in C. elegans
Source: Nutrients. 2020 Feb 14;12(2):486. doi: 10.3390/nu12020486 (PMC7071149; doi:10.3390/nu12020486)
Supplement: Supplementary file 1 [file nutrients-12-00486-s001.pdf]

Table S1. Differentially expressed genes between CR *vs.* C and S *vs.* C in brain cortex.

Transcript ID	gene_assignment	Gene Symbol	p-value (CRvsC)	Fold-Change (CRvsC)	p-value(SvsC)	Fold-Change(SvsC)
17249922	---		1.45E-06	1.23	1.62E-03	1.12
17445634	NM_198666 // Gm9758 // predicted gene 9758 // 5 A1|5 // 381714 /// ENSMUST00000154544 /	Gm9758	2.48E-06	-1.73	5.31E-04	-1.43
17532409	NM_010012 // Cyp8b1 // cytochrome P450, family 8, subfamily b, polypeptide 1 // 9 F4|9	Cyp8b1	4.23E-06	-1.24	3.71E-03	-1.12
17481680	NR_028079 // Snora3 // small nucleolar RNA, H/ACA box 3 // 7|7 // 100302499 /// ENSMUST	Snora3	8.39E-06	-1.91	9.28E-06	-1.90
17205141	---		1.03E-05	-1.87	4.13E-04	-1.59
17209331	---		1.03E-05	-1.87	4.13E-04	-1.59
17420434	---		1.15E-05	1.37	1.74E-04	1.29
17339079	---		1.18E-05	-1.42	3.96E-04	-1.30
17340397	NM_026086 // Nanp // N-acetylneuraminic acid phosphatase // 2|2 H1 // 67311 /// ENSMUST	Nanp	2.30E-05	1.40	9.02E-06	1.43
17308800	NM_025384 // Dnajc15 // DnaJ (Hsp40) homolog, subfamily C, member 15 // 14 D3|14 // 661	Dnajc15	2.36E-05	1.23	4.55E-04	1.17
17248871	---		2.43E-05	-1.44	7.40E-03	-1.23
17339395	NM_010867 // Myom1 // myomesin 1 // 17 E1.3|17 // 17929 /// NM_001083934 // Myom1 // my	Myom1	2.58E-05	-1.24	1.82E-02	-1.11
17512776	NR_002928 // Gm1943 // WD repeat domain 70 pseudogene // 8 D3|8 // 384864 /// NR_002928	Gm1943	2.71E-05	1.18	1.19E-02	1.09
17418252	NM_017475 // Rragc // Ras-related GTP binding C // 4|4 D // 54170 /// ENSMUST0000003039	Rragc	2.88E-05	1.26	1.68E-03	1.17

172 443 93	---		3.16E -05	-1.20	1.83 E-04	-1.17
174 393 65	---		3.30E -05	-1.39	1.54 E-02	-1.19
174 031 26	ENSMUST00000005964 // Adh5 // alcohol dehydrogenase 5 (class III), chi polypeptide // 3	Adh5	3.74E -05	1.36	1.91 E-04	1.31
174 475 40	NR_045505 // 4933401P06Rik // RIKEN cDNA 4933401P06 gene // 5|5 // 71054	49334 01P06 Rik	4.35E -05	-1.19	1.46 E-04	-1.17
172 557 62	NM_025927 // Mrpl45 // mitochondrial ribosomal protein L45 // 11 D|11 // 67036 /// ENSM	Mrpl4 5	4.86E -05	1.30	7.47 E-03	1.17
172 226 18	NR_027826 // 2610017I09Rik // RIKEN cDNA 2610017I09 gene // 1 B|1 // 66297 /// BC058417	26100 17I09 Rik	5.02E -05	1.35	1.36 E-03	1.25
172 969 73	XM_001475932 // Gm3187 // predicted gene 3187 // 14 A1|14 // 100041184	Gm31 87	5.03E -05	-1.48	3.67 E-04	-1.39
173 035 93	NM_146052 // Lrrc3b // leucine rich repeat containing 3B // 14 A2|14 // 218763 /// ENSM	Lrrc3b	5.85E -05	1.21	9.58 E-03	1.12
173 564 01	NM_027236 // Eif1ad // eukaryotic translation initiation factor 1A domain containing //	Eif1ad	5.99E -05	1.26	5.85 E-03	1.16
172 630 50	---		6.18E -05	-1.20	2.94 E-03	-1.13
174 182 57	---		6.21E -05	1.56	3.50 E-04	1.46
174 798 26	---		6.29E -05	-1.29	3.19 E-03	-1.19
172 691 88	---		6.43E -05	-1.25	1.92 E-03	-1.18
173 722 42	ENSMUST00000111920 // Plekha3 // pleckstrin homology domain-containing, family A (phosp	Plekha 3	6.49E -05	1.22	3.99 E-04	1.18
173 428 68	ENSMUST00000114792 // Fkbp5 // FK506 binding protein 5 // 17 A3.3|17 13.0 cM // 14229 /	Fkbp5	6.76E -05	1.34	5.85 E-06	1.42
172 692 12	---		7.79E -05	-1.15	9.88 E-05	-1.15
174 811 27	NM_147101 // Olfr549 // olfactory receptor 549 // 7 E3|7 // 259105 /// ENSMUST000000982	Olfr54 9	7.92E -05	-1.25	1.74 E-04	-1.23

174 769 46	---		8.13E -05	-1.23	1.15 E-04	-1.22
173 152 19	NM_025487 // 1700011A15Rik // RIKEN cDNA 1700011A15 gene // 15 F2|15 // 66322 /// ENSMU	17000 11A15 Rik	8.48E -05	-1.28	1.74 E-05	-1.33
172 474 58	NM_178909 // Wdr92 // WD repeat domain 92 // 11 A2|11 // 103784 /// ENSMUST00000046955	Wdr9 2	8.62E -05	1.27	6.91 E-03	1.16
174 056 12	NM_001104614 // Vmn2r3 // vomeronasal 2, receptor 3 // 3 E1|3 // 637004 /// ENSMUST0000	Vmn2r 3	9.00E -05	-1.29	3.04 E-03	-1.19
172 994 14	NR_003630 // Gm6498 // glyceraldehyde-3-phosphate dehydrogenase pseudogene // 14 C1|14	Gm64 98	9.42E -05	-1.32	2.13 E-02	-1.16
172 343 45	NR_033602 // Gm5779 // ribosomal protein, large, P0 pseudogene // 10 C1|10 // 544707 //	Gm57 79	9.48E -05	-1.34	9.04 E-04	-1.27
175 174 04	NM_021422 // Dnaja4 // DnaJ (Hsp40) homolog, subfamily A, member 4 // 9 A5.3|9 // 58233	Dnaja 4	9.76E -05	1.27	1.06 E-02	1.15
174 790 27	NM_001164201 // Lass3 // LAG1 homolog, ceramide synthase 3 // 7 C|7 // 545975 /// ENSMU	Lass3	1.03E -04	-1.19	7.99 E-03	-1.11
174 826 78	---		1.09E -04	-1.21	7.84 E-04	-1.17
174 296 09	XM_987080 // Gm10573 // predicted gene 10573 // 4 D2.2|4 // 666931 /// XM_987080 // Gm1	Gm10 573	1.10E -04	-1.17	1.09 E-02	-1.10
174 296 05	ENSMUST00000106265 // Gm12887 // predicted gene 12887 // 4 D2.2|4 // 666927 /// ENSMUST	Gm12 887	1.10E -04	-1.17	1.09 E-02	-1.10
172 808 42	ENSMUST00000001672 // Ifrd1 // interferon-related developmental regulator 1 // 12 B1|12	Ifrd1	1.11E -04	1.25	3.27 E-05	1.28
174 813 09	NM_146813 // Olfr651 // olfactory receptor 651 // 7 E3|7 // 258809 /// ENSMUST000000981	Olfr65 1	1.12E -04	-1.34	2.43 E-03	-1.24
172 499 23	---		1.13E -04	1.42	8.88 E-03	1.24
172 739 48	ENSMUST00000020911 // Sdc1 // syndecan 1 // 12 A1.1|12 1.0 cM // 20969 /// NM_011519 //	Sdc1	1.14E -04	-1.18	4.18 E-03	-1.12
172 804 79	---		1.14E -04	-1.28	1.31 E-02	-1.15
175 500 04	---		1.14E -04	-1.28	1.31 E-02	-1.15

172120 09	---		1.15E -04	-1.32	8.94 E-03	-1.19
175430 82	NR_036689 // Gm41 // melanoma antigen, family B, 4 pseudogene // X C1|X // 245502	Gm41	1.17E -04	-1.25	1.33 E-02	-1.14
174488 21	NM_026950 // Ociad2 // OCIA domain containing 2 // 5|5 D // 433904 /// ENSMUST000000871	Ociad 2	1.18E -04	1.29	3.01 E-03	1.20
174664 26	NM_001081301 // Olfr455 // olfactory receptor 455 // 6 B2.1|6 // 546896 /// ENSMUST0000	Olfr45 5	1.20E -04	-1.16	1.40 E-03	-1.13
174157 71	---		1.22E -04	-1.17	2.72 E-03	-1.12
175162 22	NM_146782 // Olfr921 // olfactory receptor 921 // 9 A5|9 // 258778 /// ENSMUST000000621	Olfr92 1	1.26E -04	-1.19	4.69 E-05	-1.21
173880 06	NM_010990 // Olfr48 // olfactory receptor 48 // 2 E1|2 // 18347 /// ENSMUST00000099762	Olfr48	1.30E -04	-1.41	4.35 E-03	-1.27
174598 95	---		1.31E -04	-1.22	1.59 E-03	-1.17
172729 39	NM_001085498 // Chmp6 // charged multivesicular body protein 6 // 11 E2|11 // 208092 //	Chmp 6	1.36E -04	-1.25	4.11 E-03	-1.17
174858 91	ENSMUST00000086363 // Tmem150b // transmembrane protein 150B // 7 A1|7 // 330460 /// NM	Tmem 150b	1.36E -04	-1.13	1.36 E-02	-1.08
175348 92	NM_030139 // Zfp449 // zinc finger protein 449 // X|X A4 // 78619 /// ENSMUST0000010156	Zfp44 9	1.37E -04	1.31	1.13 E-03	1.25
174182 56	---		1.42E -04	1.32	1.30 E-02	1.18
174185 89	NM_011902 // Tekt2 // tektin 2 // 4 D2.2|4 // 24084 /// ENSMUST00000102616 // Tekt2 //	Tekt2	1.42E -04	-1.21	2.71 E-04	-1.20
172631 93	NM_029283 // Fam183b // family with sequence similarity 183, member B // 11 B1.3|11 //	Fam1 83b	1.43E -04	1.38	2.98 E-03	1.27
174070 93	NM_020517 // Lenep // lens epithelial protein // 3 F1|3 44.5 cM // 57275 /// ENSMUST000	Lenep	1.43E -04	1.26	1.90 E-03	1.19
175147 32	NM_030261 // Sesn3 // sestrin 3 // 9|9 A3 // 75747 /// ENSMUST00000034507 // Sesn3 // s	Sesn3	1.46E -04	1.23	2.10 E-04	1.22
175508 37	---		1.56E -04	-1.24	2.26 E-04	-1.23

173 215 59	---		1.60E -04	-1.49	4.38 E-03	-1.33
173 091 13	NM_029680 // 1700110M21Rik // RIKEN cDNA 1700110M21 gene // 14 E2.3|14 // 76627 /// ENS	17001 10M2 1Rik	1.61E -04	-1.16	1.60 E-02	-1.09
175 177 70	NM_001136059 // Cyp1a1 // cytochrome P450, family 1, subfamily a, polypeptide 1 // 9 B|	Cyp1a 1	1.61E -04	-1.23	1.60 E-03	-1.18
173 681 54	NM_178036 // Lcn10 // lipocalin 10 // 2 A3|2 // 332578 /// ENSMUST00000058912 // Lcn10	Lcn10	1.61E -04	-1.21	3.52 E-02	-1.10
173 188 75	---		1.65E -04	1.78	4.24 E-02	1.32
175 466 20	XM_003689069 // LOC100862076 // uncharacterized LOC100862076 // --- // 100862076 /// XM	LOC10 08620 76	1.70E -04	-1.12	3.87 E-03	-1.09
173 353 52	NM_001034871 // Gm749 // predicted gene 749 // 17 A3.3|17 // 328788 /// ENSMUST00000025	Gm74 9	1.72E -04	-1.23	2.66 E-03	-1.17
174 559 86	NR_033518 // Gm16039 // predicted gene 16039 // 6 A1|6 // 100036521	Gm16 039	1.73E -04	1.22	5.78 E-04	1.20
175 446 23	---		1.78E -04	-1.36	2.47 E-03	-1.26
174 271 04	NM_008336 // Ifnab // interferon alpha B // 4 C4|4 // 15974 /// ENSMUST00000105146 // I	Ifnab	1.78E -04	-1.22	8.32 E-03	-1.14
173 718 70	---		1.83E -04	-1.29	3.35 E-04	-1.27
174 715 10	NM_199034 // 4922502D21Rik // RIKEN cDNA 4922502D21 gene // 6 F3|6 // 381816 /// ENSMUS	49225 02D21 Rik	1.91E -04	-1.21	3.64 E-03	-1.15
172 870 33	---		1.93E -04	-1.24	4.74 E-02	-1.11
173 878 48	ENSMUST00000099815 // Olfr1197 // olfactory receptor 1197 // 2 E1|2 // 433449 /// NM_00	Olfr11 97	1.96E -04	-1.16	8.67 E-03	-1.10
172 570 55	---		1.96E -04	1.36	5.13 E-03	1.24
173 076 23	NM_007549 // Blk // B lymphoid kinase // 14 D1|14 28.0 cM // 12143 /// ENSMUST000000145	Blk	2.01E -04	-1.27	1.52 E-02	-1.15
172 014 49	---		2.08E -04	-1.32	7.07 E-03	-1.21

175000 85	AK042983 // A730045E13Rik // RIKEN cDNA A730045E13 gene // 8 A2|8 // 208924 /// XR_1079	A730045E13 Rik	2.12E -04	-1.19	8.97 E-05	-1.20
173665 33	---		2.13E -04	-1.18	8.48 E-04	-1.15
172575 64	---		2.18E -04	1.47	1.22 E-02	1.27
172691 94	---		2.19E -04	-1.29	5.97 E-03	-1.19
174208 17	---		2.19E -04	-1.13	7.84 E-04	-1.11
174696 34	---		2.21E -04	-1.42	4.89 E-04	-1.39
173769 17	NM_027093 // 2310003L22Rik // RIKEN cDNA 2310003L22 gene // 2|2 G3 // 69487 /// ENSMUST	2310003L22 Rik	2.29E -04	1.24	9.30 E-05	1.27
172337 67	NR_039568 // Mir5108 // microRNA 5108 // 10|10 32.28 cM // 100628583	Mir5108	2.34E -04	-1.26	1.06 E-02	-1.16
173201 00	NM_011105 // Pkdrej // polycystic kidney disease (polycystin) and REJ (sperm receptor f	Pkdrej	2.39E -04	-1.14	1.57 E-03	-1.11
172903 59	---		2.40E -04	-1.18	5.15 E-05	-1.21
175351 93	AK006615 // 1700036O09Rik // RIKEN cDNA 1700036O09 gene // X|X // 73262	1700036O09 Rik	2.43E -04	-1.20	1.67 E-03	-1.16
175363 53	NM_001034907 // Zc3h12b // zinc finger CCCH-type containing 12B // X C3|X // 547176 ///	Zc3h12b	2.45E -04	1.23	5.10 E-04	1.21
174081 82	NM_146275 // Olfr1402 // olfactory receptor 1402 // 3 F2-F3|3 // 258272 /// ENSMUST0000	Olfr1402	2.48E -04	-1.33	1.12 E-03	-1.28
175107 01	NM_009033 // Rbmxl1 // RNA binding motif protein, X linked-like-1 // 8 C1|8 // 19656 //	Rbmxl1	2.51E -04	1.23	8.09 E-03	1.15
172877 72	NM_028281 // Pcbd2 // pterin 4 alpha carbinolamine dehydratase/dimerization cofactor of	Pcbd2	2.52E -04	1.26	1.95 E-03	1.21
172357 46	---		2.56E -04	-1.28	6.18 E-05	-1.32
172832 70	NM_153587 // Rps6ka5 // ribosomal protein S6 kinase, polypeptide 5 // 12 E|12 // 73086	Rps6ka5	2.57E -04	1.29	7.54 E-04	1.25

17289691	NR_045900 // 1700006H21Rik // RIKEN cDNA 1700006H21 gene // 13|13 // 72257	1700006H21Rik	2.60E-04	-1.28	4.63E-02	-1.13
17225364	NR_040482 // C030007H22Rik // RIKEN cDNA C030007H22 gene // 1|1 // 77331	C030007H22Rik	2.63E-04	-1.19	8.12E-03	-1.12
17260806	---		2.64E-04	-1.38	1.25E-02	-1.22
17330799	NR_015373 // Gm5485 // predicted gene 5485 // 16 B5|16 // 433023	Gm5485	2.75E-04	-1.21	5.83E-03	-1.15
17339172	---		2.81E-04	-1.67	1.37E-03	-1.55
17495566	ENSMUST00000044195 // Tmc7 // transmembrane channel-like gene family 7 // 7 F2|7 // 209	Tmc7	2.82E-04	1.30	3.75E-03	1.22
17269870	ENSMUST00000070395 // Ptges3l // prostaglandin E synthase 3 (cytosolic)-like // 11 D|11	Ptges3l	2.84E-04	1.20	4.18E-02	1.09
17320686	---		2.87E-04	-1.23	1.55E-03	-1.19
17441063	BC031162 // 2210016L21Rik // RIKEN cDNA 2210016L21 gene // 5 F|5 // 72357 /// ENSMUST00	2210016L21Rik	2.92E-04	1.20	2.86E-02	1.10
17487659	NM_001037248 // Gm4567 // predicted gene 4567 // 7 A3|7 // 100043645 /// NM_001201364 /	Gm4567	2.92E-04	-1.32	1.32E-02	-1.19
17318109	---		2.93E-04	-1.58	6.58E-04	-1.53
17202381	---		2.95E-04	1.33	2.65E-03	1.25
17527982	NR_039595 // Mir5133 // microRNA 5133 // 9|9 33.59 cM // 100628602 /// AF325532 // Glce	Mir5133	2.95E-04	-1.20	4.35E-03	-1.15
17285829	ENSMUST00000045301 // Hist1h1d // histone cluster 1, H1d // 13|13 A2-A3 // 14957 /// NM	Hist1h1d	2.96E-04	-1.29	2.74E-03	-1.22
17365953	NR_040316 // B230217O12Rik // RIKEN cDNA B230217O12 gene // 19|19 // 320879	B230217O12Rik	2.99E-04	1.15	1.60E-03	1.13
17316493	---		3.02E-04	1.24	1.00E-03	1.21
17220188	BC031781 // BC031781 // cDNA sequence BC031781 // 1 H4|1 // 208768 /// ENSMUST000000380	BC031781	3.02E-04	1.23	4.81E-03	1.17

175 085 66	---		3.07E -04	-1.85	2.12 E-04	-1.89
174 771 01	ENSMUST00000070518 // Nkg7 // natural killer cell group 7 sequence // 7|7 B2 // 72310 /	Nkg7	3.09E -04	-1.17	1.80 E-02	-1.10
172 090 57	---		3.12E -04	1.72	4.55 E-03	1.50
172 982 92	NM_011849 // Nek4 // NIMA (never in mitosis gene a)-related expressed kinase 4 // 14 B|	Nek4	3.14E -04	1.21	1.50 E-03	1.18
172 953 46	NM_178918 // Utp15 // UTP15, U3 small nucleolar ribonucleoprotein, homolog (yeast) // 1	Utp15	3.16E -04	1.22	8.23 E-04	1.20
172 034 03	---		3.18E -04	1.42	1.05 E-03	1.37
172 623 57	NM_172557 // Rufy1 // RUN and FYVE domain containing 1 // 11 B1.3|11 // 216724 /// ENSM	Rufy1	3.21E -04	1.23	4.61 E-04	1.22
173 226 97	AK140616 // Gm15983 // predicted gene 15983 // --- // 100294714	Gm15 983	3.27E -04	-1.18	7.11 E-03	-1.12
175 198 88	NM_022986 // Irak1bp1 // interleukin-1 receptor-associated kinase 1 binding protein 1 /	Irak1b p1	3.28E -04	1.15	9.97 E-05	1.17
174 593 03	---		3.30E -04	-1.25	3.58 E-02	-1.13
174 674 56	---		3.30E -04	-1.35	1.22 E-03	-1.30
174 397 64	---		3.31E -04	-1.39	1.12 E-04	-1.44
175 479 96	XR_141823 // LOC100862610 // uncharacterized LOC100862610 // --- // 100862610	LOC10 08626 10	3.32E -04	1.54	5.09 E-03	1.37
172 630 33	NM_001168668 // Fam114a2 // family with sequence similarity 114, member A2 // 11|11 B2	Fam1 14a2	3.37E -04	1.24	3.77 E-03	1.18
175 377 68	XM_885719 // Pramel // preferentially expressed antigen in melanoma-like // X F1|X // 6	Prame l	3.41E -04	-1.18	1.47 E-02	-1.11
173 384 34	ENSMUST00000167180 // AI314976 // expressed sequence AI314976 // 17 C|17 // 106821 ///	AI314 976	3.45E -04	1.20	4.20 E-03	1.15
172 562 62	---		3.49E -04	-1.35	4.29 E-03	-1.25

175 240 49	NM_177905 // Piwil4 // piwi-like homolog 4 (Drosophila) // 9 A2|9 // 330890 /// ENSMUST	Piwil4	3.54E -04	-1.23	4.27 E-05	-1.28
174 877 37	NM_133743 // Lypd3 // Ly6/Plaur domain containing 3 // 7 A3|7 // 72434 /// ENSMUST00000	Lypd3	3.54E -04	-1.19	6.31 E-04	-1.17
173 726 21	ENSMUST00000121114 // Slc43a1 // solute carrier family 43, member 1 // 2|2 E1 // 72401	Slc43a 1	3.56E -04	-1.13	7.97 E-04	-1.12
175 066 27	NM_008559 // Mc1r // melanocortin 1 receptor // 8 E1|8 68.0 cM // 17199 /// ENSMUST0000	Mc1r	3.59E -04	-1.21	1.39 E-03	-1.18
173 002 19	---		3.60E -04	-1.33	1.61 E-03	-1.28
173 397 06	NM_152817 // Ttc27 // tetratricopeptide repeat domain 27 // 17 E2|17 // 74196 /// ENSMU	Ttc27	3.67E -04	1.24	5.90 E-03	1.17
172 921 39	NM_001081059 // Ccdc90a // coiled-coil domain containing 90A // 13 A4|13 // 76137 /// E	Ccdc9 0a	3.67E -04	1.22	1.43 E-02	1.13
174 811 63	NM_146360 // Olfr574 // olfactory receptor 574 // 7 E3|7 // 258357 /// ENSMUST000000529	Olfr57 4	3.72E -04	-1.28	1.13 E-03	-1.25
173 130 08	NM_028634 // Cby1 // chibby homolog 1 (Drosophila) // 15 E2|15 // 73739 /// ENSMUST0000	Cby1	3.72E -04	1.31	3.03 E-03	1.24
174 263 40	BC048618 // 1700018C11Rik // RIKEN cDNA 1700018C11 gene // 4 C1|4 // 75524 /// ENSMUST0	17000 18C11 Rik	3.73E -04	-1.18	4.56 E-04	-1.18
173 256 19	NM_026407 // Tmem39a // transmembrane protein 39a // 16|16 B4 // 67846 /// ENSMUST00000	Tmem 39a	3.73E -04	1.31	2.20 E-03	1.25
175 195 79	NM_010295 // Gclc // glutamate-cysteine ligase, catalytic subunit // 9 D-E|9 42.0 cM //	Gclc	3.79E -04	1.14	8.93 E-04	1.13
173 271 91	---		3.79E -04	-1.23	2.83 E-05	-1.30
175 008 08	---		3.84E -04	-1.41	3.16 E-03	-1.31
173 001 81	X01134 // Trav9d-3 // T cell receptor alpha variable 9D-3 // 14 C2|14 // 100038850	Trav9 d-3	3.85E -04	-1.54	6.59 E-04	-1.51
174 698 64	NM_026894 // 1500001M20Rik // RIKEN cDNA 1500001M20 gene // 6 E3|6 // 68971 /// ENSMUST	15000 01M2 0Rik	3.90E -04	1.13	1.77 E-03	1.11
174 536 75	---		3.92E -04	-1.16	1.19 E-03	-1.14

17375454	NM_172980 // Slc28a2 // solute carrier family 28 (sodium-coupled nucleoside transporter	Slc28a2	3.93E-04	-1.24	3.56E-02	-1.12
17245034	NM_024454 // Rab21 // RAB21, member RAS oncogene family // 10|10 D1 // 216344 /// ENSMU	Rab21	3.93E-04	1.25	9.62E-04	1.23
17494822	NM_175532 // Nlrp10 // NLR family, pyrin domain containing 10 // 7 E3|7 // 244202 /// E	Nlrp10	3.97E-04	-1.28	3.84E-03	-1.21
17455574	---		3.98E-04	-1.33	1.55E-02	-1.20
17235947	NM_026579 // D10Wsu102e // DNA segment, Chr 10, Wayne State University 102, expressed /	D10Wsu102e	4.00E-04	1.18	3.05E-03	1.14
17446750	---		4.00E-04	-1.42	2.80E-02	-1.22
17427623	NR_040708 // E130102H24Rik // RIKEN cDNA E130102H24 gene // 4 C6|4 // 77866 /// NR_0295	E130102H24Rik	4.01E-04	1.23	3.03E-04	1.24
17219974	---		4.02E-04	1.47	1.44E-02	1.28
17383347	AK019269 // 2810430I11Rik // RIKEN cDNA 2810430I11 gene // --- // 641385	2810430I11Rik	4.13E-04	-1.19	2.57E-04	-1.20
17428249	---		4.14E-04	-1.31	8.70E-03	-1.21
17231188	NM_145415 // Diexf // digestive organ expansion factor homolog (zebrafish) // 1 H6|1 //	Diexf	4.14E-04	1.32	3.68E-02	1.16
17379482	ENSMUST00000109334 // Wfdc10 // WAP four-disulfide core domain 10 // 2 H3|2 // 629756 /	Wfdc10	4.14E-04	-1.18	7.09E-03	-1.13
17378575	BC140978 // 4921517L17Rik // RIKEN cDNA 4921517L17 gene // 2 H2|2 // 70873 /// ENSMUST0	4921517L17Rik	4.21E-04	1.17	5.10E-03	1.13
17206643	---		4.21E-04	1.99	2.13E-03	1.78
17474266	---		4.22E-04	-1.28	1.22E-03	-1.25
17508164	---		4.25E-04	1.37	2.01E-02	1.21
17379978	ENSMUST00000099073 // Fam65c // family with sequence similarity 65, member C // 2 H3|2	Fam65c	4.36E-04	-1.16	2.04E-03	-1.13

174 001 24	NM_019414 // Selenbp2 // selenium binding protein 2 // 3 F2.1|3 43.26 cM // 20342 /// E	Selenb p2	4.45E -04	-1.19	1.65 E-05	-1.26
173 949 95	NR_040509 // 1700028P15Rik // RIKEN cDNA 1700028P15 gene // 2|2 // 75655	17000 28P15 Rik	4.47E -04	-1.23	1.02 E-03	-1.21
173 579 30	NM_146636 // Olfr1487 // olfactory receptor 1487 // 19 C1|19 // 258629 /// ENSMUST00000	Olfr14 87	4.48E -04	-1.34	1.17 E-02	-1.22
173 243 03	---		4.54E -04	-1.36	7.70 E-04	-1.34
173 190 22	NM_028331 // C1qtnf6 // C1q and tumor necrosis factor related protein 6 // 15 E1|15 //	C1qtnf 6	4.58E -04	-1.23	2.21 E-02	-1.13
172 075 63	---		4.59E -04	-1.26	8.87 E-03	-1.18
172 086 83	---		4.61E -04	1.40	1.38 E-02	1.25
175 285 52	---		4.61E -04	-1.33	1.86 E-02	-1.20
172 786 88	NR_030438 // Mir673 // microRNA 673 // 12|12 // 751547	Mir67 3	4.61E -04	-1.27	1.73 E-03	-1.23
174 768 60	XM_001481155 // Gm10166 // predicted pseudogene 10166 // 7 B2|7 // 100043872	Gm10 166	4.64E -04	-1.25	4.96 E-03	-1.19
172 033 73	---		4.65E -04	-1.38	2.56 E-03	-1.31
173 865 69	NM_001113246 // Chn1 // chimerin (chimaerin) 1 // 2 C3|2 // 108699 /// ENSMUST000001120	Chn1	4.67E -04	1.19	9.85 E-03	1.13
172 997 73	---		4.71E -04	-1.19	1.26 E-02	-1.13
172 365 31	NM_008517 // Lta4h // leukotriene A4 hydrolase // 10|10 C3 // 16993 /// ENSMUST00000016	Lta4h	4.71E -04	1.22	4.15 E-02	1.11
174 182 62	---		4.73E -04	1.26	2.53 E-02	1.15
174 014 18	NM_027025 // Adora3 // adenosine A3 receptor // 3 F2.2|3 55.7 cM // 11542 /// ENSMUST00	Adora 3	4.77E -04	-1.14	4.15 E-04	-1.14
173 629 86	NM_013516 // Ms4a2 // membrane-spanning 4-domains, subfamily A, member 2 // 19 A|19 8.0	Ms4a2	4.82E -04	-1.14	8.44 E-03	-1.10

17287977	NR_036449 // Gm5084 // predicted gene 5084 // 13 B2|13 // 100503759	Gm5084	4.85E-04	-1.15	1.87E-03	-1.13
17420430	---		4.85E-04	1.35	2.85E-04	1.37
17474542	---		4.89E-04	-1.22	1.75E-03	-1.19
17311157	NM_025494 // Atp6v1c1 // ATPase, H+ transporting, lysosomal V1 subunit C1 // 15|15 C //	Atp6v1c1	4.89E-04	1.29	6.39E-03	1.21
17488176	XR_107882 // Gm15567 // predicted gene 15567 // 7|7 15.86 cM // 100503685	Gm15567	4.92E-04	-1.24	2.68E-02	-1.13
17253937	NM_139228 // Rhbdl3 // rhomboid, veinlet-like 3 (Drosophila) // 11 B5|11 // 246104 ///	Rhbdl3	4.93E-04	1.21	3.67E-02	1.11
17375308	NM_030234 // Wdr76 // WD repeat domain 76 // 2|2 F1 // 241627 /// ENSMUST00000028676 //	Wdr76	4.94E-04	1.28	2.40E-03	1.23
17354502	NR_045360 // 2210409D07Rik // RIKEN cDNA 2210409D07 gene // 18 D3|18 // 70157	2210409D07Rik	4.95E-04	-1.13	9.82E-03	-1.09
17343289	NR_027316 // Pdxk-ps // pyridoxal (pyridoxine, vitamin B6) kinase, pseudogene // 17 B1|	Pdxk-ps	5.06E-04	-1.15	2.79E-04	-1.16
17486944	NR_003247 // Ceacam-ps1 // carcinoembryonic antigen-related cell adhesion molecule pseu	Ceacam-ps1	5.12E-04	-1.22	2.87E-03	-1.18
17539411	---		5.17E-04	-1.25	8.84E-03	-1.17
17315789	NM_001081402 // Wdr70 // WD repeat domain 70 // 15 A1|15 // 545085 /// ENSMUST000000457	Wdr70	5.18E-04	1.23	1.37E-03	1.21
17203405	---		5.18E-04	1.71	1.40E-03	1.62
17211533	NM_153601 // Lgsn // lengsin, lens protein with glutamine synthetase domain // 1 B|1 //	Lgsn	5.20E-04	-1.15	3.41E-04	-1.15
17342507	NR_028042 // Gm10012 // cytochrome c oxidase, subunit VIIc pseudogene // 17 A3.3|17 //	Gm10012	5.21E-04	-1.32	4.91E-02	-1.15
17394578	---		5.22E-04	-1.21	3.84E-04	-1.21
17356642	ENSMUST00000044451 // Naaladl1 // N-acetylated alpha-linked acidic dipeptidase-like 1 /	Naaladl1	5.27E-04	-1.17	1.73E-03	-1.15

17269246	NM_027087 // Krtap4-13 // keratin associated protein 4-13 // 11 D|11 // 69464 /// ENSMU	Krtap4-13	5.28E-04	-1.19	5.56E-04	-1.19
17349460	AK029153 // 2010110K18Rik // RIKEN cDNA 2010110K18 gene // 18|18 // 100503082 /// AK008	2010110K18Rik	5.30E-04	-1.17	3.72E-02	-1.09
17210643	---		5.33E-04	1.42	1.18E-02	1.27
17303757	NM_134081 // Dnajc9 // DnaJ (Hsp40) homolog, subfamily C, member 9 // 14 A3|14 // 10867	Dnajc9	5.34E-04	1.25	6.83E-04	1.24
17361090	NM_144869 // BC021614 // cDNA sequence BC021614 // 19 A|19 // 225884 /// ENSMUST0000007	BC021614	5.37E-04	-1.21	1.42E-03	-1.19
17204721	---		5.56E-04	2.15	1.47E-02	1.66
17293237	NM_027307 // Golm1 // golgi membrane protein 1 // 13|13 B3 // 105348 /// ENSMUST0000009	Golm1	5.56E-04	1.29	2.12E-03	1.24
17203533	---		5.61E-04	-1.33	1.83E-02	-1.20
17504372	---		5.64E-04	-1.43	5.54E-03	-1.32
17439307	ENSMUST00000161225 // Gm3259 // predicted gene 3259 // 5 E3|5 // 100041296 /// ENSMUST0	Gm3259	5.64E-04	-1.28	3.51E-02	-1.15
17299922	---		5.64E-04	-1.15	1.40E-02	-1.10
17292654	ENSMUST00000021920 // Sptlc1 // serine palmitoyltransferase, long chain base subunit 1	Sptlc1	5.69E-04	1.19	4.12E-03	1.15
17500060	NR_030254 // Mir486 // microRNA 486 // 8|8 // 723876 /// NR_037293 // Mir3107 // microR	Mir486	5.81E-04	-1.19	2.24E-04	-1.21
17230602	NM_175127 // Fbxo28 // F-box protein 28 // 1 H5|1 98.7 cM // 67948 /// ENSMUST000000514	Fbxo28	5.91E-04	1.24	7.07E-03	1.17
17358128	---		5.93E-04	-1.17	4.47E-04	-1.18
17430503	NM_008468 // Kpna6 // karyopherin (importin) alpha 6 // 4|4 D2.3 // 16650 /// ENSMUST00	Kpna6	5.94E-04	-1.13	1.49E-02	-1.09
17231855	NM_053008 // Olig3 // oligodendrocyte transcription factor 3 // 10 A3|10 // 94222 /// E	Olig3	5.99E-04	-1.17	1.18E-02	-1.12

174 542 16	ENSMUST00000165640 // Zfp113 // zinc finger protein 113 // 5|5 G1 // 56314 /// NM_01974	Zfp11 3	6.00E -04	1.17	1.61 E-03	1.15
173 082 42	NM_001253736 // Pdlim2 // PDZ and LIM domain 2 // 14 D2|14 // 213019 /// ENSMUST0000015	Pdlim 2	6.03E -04	-1.20	3.68 E-03	-1.16
174 391 31	NM_175096 // Stbd1 // starch binding domain 1 // 5 E2|5 52.0 cM // 52331 /// ENSMUST000	Stbd1	6.04E -04	-1.18	2.63 E-03	-1.15
173 599 66	---		6.30E -04	-1.30	5.92 E-04	-1.30
173 858 38	---		6.32E -04	-1.50	5.07 E-04	-1.51
175 492 84	---		6.34E -04	-1.21	6.14 E-05	-1.26
175 126 91	---		6.37E -04	1.24	1.87 E-03	1.21
174 285 29	NM_028142 // Nsun4 // NOL1/NOP2/Sun domain family, member 4 // 4 D1|4 // 72181 /// ENSM	Nsun4	6.42E -04	1.21	9.45 E-04	1.20
175 087 35	BC094602 // Tnks // tankyrase, TRF1-interacting ankyrin-related ADP-ribose polymerase /	Tnks	6.42E -04	1.42	1.35 E-03	1.38
174 426 91	NM_001081750 // Zfp664 // zinc finger protein 664 // 5 F|5 // 269704 /// ENSMUST0000011	Zfp66 4	6.47E -04	1.19	1.20 E-03	1.18
175 002 75	NM_153592 // Erlin2 // ER lipid raft associated 2 // 8 A2|8 // 244373 /// ENSMUST000000	Erlin2	6.53E -04	1.19	4.89 E-03	1.15
174 956 51	NM_027943 // Pdilt // protein disulfide isomerase-like, testis expressed // 7|7 F3 // 7	Pdilt	6.55E -04	-1.27	2.36 E-02	-1.16
173 565 56	NR_002847 // Malat1 // metastasis associated lung adenocarcinoma transcript 1 (non-codi	Malat 1	6.60E -04	-1.67	1.67 E-02	-1.40
172 604 53	NR_028586 // Gm11978 // predicted gene 11978 // 11 A1|11 // 100041311	Gm11 978	6.70E -04	-1.19	3.78 E-02	-1.10
175 026 92	NM_183096 // Ttc29 // tetratricopeptide repeat domain 29 // 8 C1|8 // 73301 /// ENSMUST	Ttc29	6.74E -04	-1.14	4.99 E-04	-1.14
174 037 31	---		6.76E -04	-1.39	5.83 E-03	-1.29
175 416 34	---		6.76E -04	-1.55	2.36 E-03	-1.46

172 799 50	NM_173417 // Kcns3 // potassium voltage-gated channel, delayed-rectifier, subfamily S,	Kcns3	6.86E -04	-1.16	2.48 E-02	-1.10
174 261 73	ENSMUST00000107454 // Rnf183 // ring finger protein 183 // 4 B3|4 // 76072 /// ENSMUST0	Rnf18 3	6.88E -04	-1.20	1.11 E-02	-1.14
173 060 54	ENSMUST00000159292 // Osgep // O-sialoglycoprotein endopeptidase // 14 C1|14 // 66246 /	Osgep	6.91E -04	1.23	1.05 E-02	1.16
175 132 75	---		6.95E -04	-1.21	9.73 E-03	-1.15
172 892 89	NM_026173 // Poc5 // POC5 centriolar protein homolog (Chlamydomonas) // 13 D1|13 // 674	Poc5	6.95E -04	1.16	7.42 E-04	1.16
174 607 85	NM_144940 // Uroc1 // urocanase domain containing 1 // 6 D1|6 // 243537 /// ENSMUST0000	Uroc1	6.98E -04	-1.13	3.10 E-04	-1.14
175 078 82	ENSMUST00000122025 // Gm15056 // predicted gene 15056 // 8 A2|8 // 100504014 /// NM_001	Gm15 056	7.02E -04	-1.25	1.93 E-02	-1.16
172 327 34	NM_001168304 // Cdk19 // cyclin-dependent kinase 19 // 10 B1|10 // 78334 /// ENSMUST000	Cdk19	7.04E -04	1.18	1.80 E-03	1.17
174 688 89	NM_011911 // Vmn1r49 // vomeronasal 1, receptor 49 // 6 D1|6 37.0 cM // 24112 /// ENSMU	Vmn1r 49	7.06E -04	-1.31	1.27 E-02	-1.21
175 483 54	NM_025431 // Llph // LLP homolog, long-term synaptic facilitation (Aplysia) // 10 D2|10	Llph	7.07E -04	1.46	6.58 E-04	1.46
173 089 63	---		7.08E -04	-1.53	4.97 E-03	-1.40
174 860 60	NM_030742 // Vmn1r63 // vomeronasal 1 receptor 63 // 7 A1|7 // 81017 /// NM_030741 // V	Vmn1r 63	7.11E -04	-1.17	2.07 E-02	-1.11
174 985 46	NM_030742 // Vmn1r63 // vomeronasal 1 receptor 63 // 7 A1|7 // 81017 /// NM_030741 // V	Vmn1r 63	7.11E -04	-1.17	2.07 E-02	-1.11
175 486 99	AK136274 // A930006D01Rik // RIKEN cDNA A930006D01 gene // 8|8 // 68163 /// XR_106168 /	A9300 06D01 Rik	7.15E -04	-1.17	1.34 E-02	-1.11
174 477 37	NM_027295 // Rab28 // RAB28, member RAS oncogene family // 5|5 B2 // 100972 /// ENSMUST	Rab28	7.16E -04	1.34	1.05 E-03	1.32
172 999 16	---		7.18E -04	-1.16	6.46 E-03	-1.12
173 542 90	NM_026217 // Atg12 // autophagy related 12 // 18 C|18 // 67526 /// ENSMUST00000035648 /	Atg12	7.23E -04	1.30	2.01 E-03	1.27

172465 20	NM_023743 // Eif4enif1 // eukaryotic translation initiation factor 4E nuclear import fa	Eif4en if1	7.32E -04	1.28	4.66 E-03	1.22
174447 70	NM_001243109 // Gm3404 // predicted gene 3404 // 5 G3|5 // 100041554 /// ENSMUST0000011	Gm34 04	7.37E -04	-1.18	7.92 E-04	-1.18
173488 95	---		7.43E -04	-1.58	2.59 E-02	-1.32
175500 84	---		7.43E -04	-1.58	2.59 E-02	-1.32
172634 48	NR_045476 // 1700007J10Rik // RIKEN cDNA 1700007J10 gene // 11|11 // 69320	17000 07J10 Rik	7.44E -04	-1.20	8.52 E-03	-1.14
173297 59	NM_007470 // Apod // apolipoprotein D // 16 B2|16 21.2 cM // 11815 /// ENSMUST000000232	Apod	7.51E -04	1.25	7.07 E-03	1.19
174490 33	NM_172146 // Ppat // phosphoribosyl pyrophosphate amidotransferase // 5 C3.3|5 // 23132	Ppat	7.56E -04	1.30	4.88 E-03	1.24
174889 45	NM_152814 // Zfp566 // zinc finger protein 566 // 7 B1|7 // 72556 /// ENSMUST0000008878	Zfp56 6	7.63E -04	1.24	3.17 E-04	1.26
175265 50	---		7.64E -04	1.41	8.51 E-03	1.29
172164 78	NM_011459 // Serpinb8 // serine (or cysteine) peptidase inhibitor, clade B, member 8 //	Serpin b8	7.67E -04	1.37	4.99 E-05	1.49
175295 57	---		7.71E -04	1.61	5.31 E-03	1.46
175295 90	---		7.71E -04	1.61	5.31 E-03	1.46
175081 63	---		7.75E -04	1.34	9.12 E-05	1.43
174749 00	NM_019941 // Zfp235 // zinc finger protein 235 // 7 A3|7 // 56525 /// ENSMUST0000005654	Zfp23 5	7.76E -04	1.19	1.07 E-02	1.13
174030 25	NM_019920 // Lamtor3 // late endosomal/lysosomal adaptor, MAPK and MTOR activator 3 //	Lamto r3	7.77E -04	1.15	5.38 E-03	1.12
173782 60	NM_029362 // Chmp4b // charged multivesicular body protein 4B // 2|2 H2 // 75608 /// EN	Chmp 4b	7.79E -04	1.29	4.92 E-03	1.23
172057 63	---		7.85E -04	1.50	9.99 E-03	1.34

17427147	NM_009877 // Cdkn2a // cyclin-dependent kinase inhibitor 2A // 4 C3-C6|4 42.7 cM // 125	Cdkn2a	7.86E-04	-1.19	8.86E-04	-1.19
17354386	NM_178686 // Cep120 // centrosomal protein 120 // 18 D1|18 // 225523 /// ENSMUST0000004	Cep120	7.87E-04	1.13	5.56E-04	1.13
17219299	NM_011070 // Pfdn2 // prefoldin 2 // 1 H3|1 92.6 cM // 18637 /// ENSMUST00000135941 //	Pfdn2	7.90E-04	1.24	1.54E-03	1.22
17443801	---		7.90E-04	-2.04	4.13E-03	-1.80
17300040	---		7.94E-04	-1.20	4.61E-03	-1.16
17207205	---		8.06E-04	1.77	5.37E-04	1.81
17487545	NM_001037248 // Gm4567 // predicted gene 4567 // 7 A3|7 // 100043645 /// NM_001037248 /	Gm4567	8.07E-04	-1.25	1.96E-02	-1.16
17254662	ENSMUST00000108021 // Ptrh2 // peptidyl-tRNA hydrolase 2 // 11 C|11 // 217057 /// ENSMU	Ptrh2	8.16E-04	1.24	2.27E-03	1.21
17285516	Z48592 // Tcrg-V6 // T cell receptor gamma, variable 6 // --- // 21640	Tcrg-V6	8.17E-04	-1.26	9.22E-03	-1.19
17375935	NM_028765 // Acoxl // acyl-Coenzyme A oxidase-like // 2 F1|2 // 74121 /// ENSMUST000000	Acoxl	8.30E-04	-1.14	2.76E-02	-1.08
17436627	---		8.30E-04	-1.51	1.42E-02	-1.33
17433328	NM_011067 // Per3 // period homolog 3 (Drosophila) // 4 E2|4 // 18628 /// ENSMUST000001	Per3	8.42E-04	1.15	1.47E-04	1.18
17408497	NM_013486 // Cd2 // CD2 antigen // 3 F2.2|3 48.2 cM // 12481 /// ENSMUST00000029456 //	Cd2	8.45E-04	-1.20	2.13E-03	-1.18
17429942	NR_015560 // 2610028E06Rik // RIKEN cDNA 2610028E06 gene // 4 D2.2|4 // 72395	2610028E06Rik	8.59E-04	-1.24	3.79E-02	-1.13
17288434	NM_029873 // 8030423J24Rik // RIKEN cDNA 8030423J24 gene // 13 C1|13 // 77166 /// ENSMU	8030423J24Rik	8.73E-04	-1.19	2.61E-03	-1.17
17373237	BC042745 // A330069E16Rik // RIKEN cDNA A330069E16 gene // 2 E1|2 // 606735	A330069E16Rik	8.76E-04	-1.11	2.49E-02	-1.07
17503557	---		8.79E-04	-1.28	1.51E-02	-1.19

175351 83	NM_198863 // Slitrk2 // SLIT and NTRK-like family, member 2 // X|X A5 // 245450 /// NM_	Slitrk2	8.82E -04	1.25	3.44 E-02	1.14
172723 75	ENSMUST00000134182 // Qrich2 // glutamine rich 2 // 11 E2|11 // 217341 /// NM_001033267	Qrich2	8.82E -04	-1.15	3.38 E-02	-1.09
175492 30	---		8.83E -04	-1.17	1.58 E-02	-1.12
174706 27	NM_019948 // Clec4e // C-type lectin domain family 4, member e // 6 F3|6 59.6 cM // 566	Clec4e	8.86E -04	-1.21	4.13 E-03	-1.17
173027 73	NM_023579 // Ipo5 // importin 5 // 14 E5|14 // 70572 /// ENSMUST00000032898 // Ipo5 //	Ipo5	8.88E -04	1.18	2.47 E-03	1.16
173467 35	NM_013933 // Vapa // vesicle-associated membrane protein, associated protein A // 17|17	Vapa	8.98E -04	1.17	9.50 E-03	1.13
174029 32	---		9.00E -04	-1.20	9.07 E-04	-1.20
175021 79	NM_172756 // Ankle1 // ankyrin repeat and LEM domain containing 1 // 8 B3.3|8 // 234396	Ankle 1	9.11E -04	-1.19	3.31 E-03	-1.16
174031 14	NM_011996 // Adh4 // alcohol dehydrogenase 4 (class II), pi polypeptide // 3|3 H2 // 26	Adh4	9.24E -04	-1.24	1.56 E-02	-1.16
172382 10	ENSMUST00000026461 // Prim1 // DNA primase, p49 subunit // 10 D3|10 77.0 cM // 19075 //	Prim1	9.37E -04	1.23	6.36 E-04	1.24
174427 01	BC053444 // Fam101a // family with sequence similarity 101, member A // 5|5 F // 73121	Fam1 01a	9.49E -04	-1.14	3.15 E-04	-1.16
173036 65	XM_003085899 // Gm9637 // predicted gene 9637 // 14 A3|14 // 675165	Gm96 37	9.58E -04	-1.12	1.30 E-02	-1.09
172658 21	NM_146709 // Olfr411 // olfactory receptor 411 // 11 B4|11 // 258704 /// ENSMUST0000005	Olfr41 1	9.65E -04	-1.19	7.46 E-03	-1.15
174618 45	---		9.67E -04	-1.17	3.14 E-03	-1.15
174854 97	NM_001033431 // Nlrp12 // NLR family, pyrin domain containing 12 // 7 A1|7 // 378425 //	Nlrp1 2	9.67E -04	-1.17	7.57 E-03	-1.13
172508 24	NM_025496 // Cdrt4 // CMT1A duplicated region transcript 4 // 11 B2-B3|11 // 66338 ///	Cdrt4	9.70E -04	-1.21	1.56 E-02	-1.14
172943 97	ENSMUST00000022063 // Ccdc127 // coiled-coil domain containing 127 // 13 C1|13 // 67433	Ccdc1 27	9.72E -04	-1.16	2.91 E-04	-1.18

17403148	NR_035481 // Mir1956 // microRNA 1956 // --- // 100316706	Mir1956	9.77E-04	-1.27	4.81E-04	-1.30
17216031	NM_145519 // Farp2 // FERM, RhoGEF and pleckstrin domain protein 2 // 1 D|1 // 227377 /	Farp2	9.79E-04	1.17	8.73E-03	1.13
17432565	NM_001085503 // Aadacl3 // arylacetamide deacetylase-like 3 // 4 E1|4 // 230883 /// ENS	Aadacl3	9.83E-04	-1.24	3.02E-03	-1.21
17498080	ENSMUST00000106074 // Krtap5-1 // keratin associated protein 5-1 // 7 F5|7 // 50774 ///	Krtap5-1	9.83E-04	-1.20	6.53E-04	-1.21
17354363	---		9.87E-04	-1.41	4.29E-03	-1.33
17464922	ENSMUST00000060442 // Gpr85 // G protein-coupled receptor 85 // 6 A1|6 // 64450 /// NM_	Gpr85	9.89E-04	1.16	8.19E-03	1.12
17482560	NM_007908 // Eef2k // eukaryotic elongation factor-2 kinase // 7|7 F3 // 13631 /// NM_0	Eef2k	9.94E-04	1.15	4.57E-04	1.17
17420446	---		9.96E-04	1.52	1.08E-02	1.36
17423461	---		1.00E-03	-1.34	1.28E-02	-1.23
17402181	NM_010171 // F3 // coagulation factor III // 3 G1|3 50.0 cM // 14066 /// ENSMUST0000002	F3	1.01E-03	1.20	6.02E-04	1.22
17441396	NM_001033311 // Vsig10 // V-set and immunoglobulin domain containing 10 // 5 F|5 // 231	Vsig10	1.01E-03	-1.22	2.17E-04	-1.25
17341705	---		1.01E-03	1.26	1.09E-02	1.18
17289239	NM_009321 // Tbca // tubulin cofactor A // 13 D1|13 // 21371 /// ENSMUST00000046644 //	Tbca	1.02E-03	1.26	5.83E-03	1.21
17533939	NM_027955 // Gmcl1l // germ cell-less homolog 1 (Drosophila)-like // X A3.1|X // 71847	Gmcl1l	1.03E-03	-1.29	1.66E-04	-1.35
17229113	NM_178883 // Gorab // golgin, RAB6-interacting // 1 H2.1|1 // 98376 /// ENSMUST00000045	Gorab	1.04E-03	1.15	7.99E-04	1.15
17457381	ENSMUST00000162554 // Ttc26 // tetratricopeptide repeat domain 26 // 6 B1|6 // 264134 /	Ttc26	1.04E-03	1.22	1.36E-02	1.15
17319539	NM_026737 // Phf5a // PHD finger protein 5A // 15 E|15 // 68479 /// ENSMUST00000023117	Phf5a	1.04E-03	1.40	2.92E-02	1.23

172 148 23	---		1.04E -03	-2.53	1.57 E-02	-1.91
173 442 89	NM_019467 // Aif1 // allograft inflammatory factor 1 // 17 B1|17 19.05 cM // 11629 ///	Aif1	1.05E -03	1.11	3.69 E-03	1.09
174 262 22	ENSMUST00000030091 // Pole3 // polymerase (DNA directed), epsilon 3 (p17 subunit) // 4|	Pole3	1.05E -03	1.16	9.04 E-03	1.12
174 278 14	NM_028209 // Ttc4 // tetratricopeptide repeat domain 4 // 4 C7|4 // 72354 /// ENSMUST00	Ttc4	1.06E -03	1.15	2.38 E-03	1.14
175 494 46	---		1.06E -03	-1.24	9.18 E-03	-1.17
175 498 34	---		1.06E -03	-1.24	9.18 E-03	-1.17
172 555 43	NR_037977 // Gm53 // predicted gene 53 // 11 D|11 // 193022	Gm53	1.06E -03	-1.15	2.17 E-04	-1.17
172 332 37	NM_001159389 // Rfx6 // regulatory factor X, 6 // 10 B3|10 // 320995 /// ENSMUST0000012	Rfx6	1.06E -03	-1.21	1.17 E-02	-1.15
172 772 70	NM_173756 // Lin52 // lin-52 homolog (C. elegans) // 12 D1|12 // 217708 /// BC120496 //	Lin52	1.07E -03	1.17	7.06 E-03	1.13
172 841 11	NR_045295 // 2810029C07Rik // RIKEN cDNA 2810029C07 gene // 12 F1|12 // 100504687	28100 29C07 Rik	1.07E -03	1.15	1.09 E-02	1.11
173 104 66	NM_001130149 // Drosha // drosha, ribonuclease type III // 15 A1|15 // 14000 /// NM_026	Drosh a	1.07E -03	1.17	3.30 E-02	1.10
172 663 15	NR_033549 // Gm11190 // predicted gene 11190 // --- // 791423	Gm11 190	1.07E -03	-1.19	6.99 E-03	-1.15
175 491 28	---		1.08E -03	-1.20	1.46 E-03	-1.20
173 154 90	ENSMUST00000055562 // Hoxc12 // homeobox C12 // 15 F3|15 57.4 cM // 15421 /// NM_010463	Hoxc1 2	1.08E -03	-1.23	1.83 E-02	-1.15
174 682 31	NR_002871 // Vax2os2 // Vax2 opposite strand transcript 2 // 6 C3|6 // 545859 /// NR_00	Vax2o s2	1.08E -03	-1.21	6.30 E-03	-1.17
174 710 73	NM_001033126 // Cd27 // CD27 antigen // 6 F3|6 60.35 cM // 21940 /// ENSMUST00000032486	Cd27	1.09E -03	-1.16	2.99 E-02	-1.10
175 483 11	AY140896 // Gm3579 // predicted gene 3579 // 4 D2.3|4 // 100041932 /// AY140896 // Gm35	Gm35 79	1.09E -03	1.58	1.87 E-02	1.36

175 483 13	AY140896 // Gm3579 // predicted gene 3579 // 4 D2.3|4 // 100041932 /// AY140896 // Gm35	Gm35 79	1.09E -03	1.58	1.87 E-02	1.36
175 486 42	AY140896 // Gm3579 // predicted gene 3579 // 4 D2.3|4 // 100041932 /// AY140896 // Gm35	Gm35 79	1.09E -03	1.58	1.87 E-02	1.36
175 486 44	AY140896 // Gm3579 // predicted gene 3579 // 4 D2.3|4 // 100041932 /// AY140896 // Gm35	Gm35 79	1.09E -03	1.58	1.87 E-02	1.36
173 837 33	NM_144885 // BC005624 // cDNA sequence BC005624 // 2 B|2 // 227707 /// ENSMUST000000282	BC005 624	1.09E -03	1.24	2.21 E-03	1.22
173 594 54	BC116839 // AI606181 // expressed sequence AI606181 // 19 C3|19 // 226118	AI606 181	1.10E -03	1.21	9.60 E-03	1.16
174 147 87	---		1.11E -03	-1.20	9.44 E-04	-1.20
172 494 83	NM_026479 // Zcchc10 // zinc finger, CCHC domain containing 10 // 11 B1.3|11 28.5 cM //	Zcchc 10	1.11E -03	1.26	2.27 E-02	1.16
174 912 76	NM_175318 // Spty2d1 // SPT2, Suppressor of Ty, domain containing 1 (S. cerevisiae) //	Spty2 d1	1.11E -03	1.21	4.52 E-02	1.12
175 148 53	ENSMUST00000061568 // Slc36a4 // solute carrier family 36 (proton/amino acid symporter)	Slc36a 4	1.12E -03	1.17	4.61 E-03	1.14
172 575 65	---		1.12E -03	2.24	1.08 E-02	1.83
175 484 64	NM_007950 // Ereg // epiregulin // 5 E1|5 // 13874 /// ENSMUST00000031324 // Ereg // ep	Ereg	1.13E -03	-1.16	1.16 E-02	-1.12
175 293 56	---		1.13E -03	-1.16	7.85 E-04	-1.17
174 584 65	NM_175098 // Ccdc126 // coiled-coil domain containing 126 // 6 B2.3|6 // 57895 /// ENSM	Ccdc1 26	1.15E -03	1.19	4.91 E-02	1.11
175 072 58	NM_183315 // Ctxn1 // cortexin 1 // 8 A1.1|8 13.0 cM // 330695 /// ENSMUST00000053252 /	Ctxn1	1.16E -03	-1.17	2.09 E-03	-1.16
174 593 27	---		1.16E -03	-1.24	3.36 E-03	-1.21
172 601 82	ENSMUST00000118076 // Urgcp // upregulator of cell proliferation // 11 A1|11 // 72046 /	Urgcp	1.16E -03	1.19	2.11 E-02	1.12
172 263 27	NM_010766 // Marco // macrophage receptor with collagenous structure // 1|1 E4-F // 171	Marco	1.16E -03	-1.17	3.83 E-02	-1.10

173076 73	NM_001081063 // Prss55 // protease, serine, 55 // 14 D1|14 // 71037 /// ENSMUST00000089	Prss55	1.16E -03	-1.18	1.26 E-02	-1.13
174674 10	---		1.16E -03	-1.22	6.04 E-03	-1.18
174979 75	NR_040337 // Gm16982 // predicted gene, 16982 // --- // 100036523	Gm16 982	1.17E -03	-1.14	4.71 E-03	-1.12
174229 75	---		1.17E -03	-1.13	4.61 E-02	-1.07
172170 58	ENSMUST00000049027 // Slc26a9 // solute carrier family 26, member 9 // 1 E4|1 // 320718	Slc26a 9	1.18E -03	-1.12	1.20 E-03	-1.12
173179 00	XM_003085972 // Gm10362 // predicted gene 10362 // 15 D3|15 // 100040929 /// XM_0014755	Gm10 362	1.19E -03	-1.15	1.43 E-04	-1.19
174204 25	NM_001122897 // Hp1bp3 // heterochromatin protein 1, binding protein 3 // 4 D3|4 // 154	Hp1bp 3	1.19E -03	1.16	1.21 E-02	1.12
174495 48	---		1.19E -03	1.28	8.79 E-05	1.37
173423 08	ENSMUST00000048054 // Chtf18 // CTF18, chromosome transmission fidelity factor 18 // 17	Chtf18	1.19E -03	-1.14	1.11 E-04	-1.18
174077 06	---		1.20E -03	1.38	3.32 E-03	1.33
172998 12	---		1.20E -03	-1.43	3.18 E-03	-1.37
173762 12	NM_177726 // Tgm6 // transglutaminase 6 // 2 F1|2 // 241636 /// ENSMUST00000028888 // T	Tgm6	1.20E -03	-1.17	7.25 E-03	-1.13
172135 70	---		1.21E -03	-1.18	7.22 E-03	-1.14
173293 45	NM_009967 // Crygs // crystallin, gamma S // 16 B1|16 16.1 cM // 12970 /// ENSMUST00000	Crygs	1.21E -03	-1.21	2.60 E-03	-1.19
173016 05	ENSMUST00000062437 // Nkx2-6 // NK2 transcription factor related, locus 6 (Drosophila)	Nkx2- 6	1.22E -03	-1.15	4.50 E-03	-1.13
172756 89	NM_025656 // Sip1 // survival of motor neuron protein interacting protein 1 // 12|12 C2	Sip1	1.22E -03	1.26	2.09 E-03	1.24
175477 71	NM_011452 // Serpinb9b // serine (or cysteine) peptidase inhibitor, clade B, member 9b	Serpin b9b	1.23E -03	-1.16	1.69 E-02	-1.11

173079 63	U92703 // Ebf2 // early B cell factor 2 // 14|14 D-E1 // 13592	Ebf2	1.23E -03	-1.20	2.19 E-02	-1.13
172103 97	---		1.24E -03	1.73	7.31 E-04	1.78
172139 53	NM_029160 // Spag16 // sperm associated antigen 16 // 1 C3|1 // 66722 /// ENSMUST000000	Spag1 6	1.24E -03	-1.20	1.49 E-02	-1.14
172207 61	---		1.24E -03	1.27	5.29 E-04	1.29
172343 47	ENSMUST00000039925 // Upb1 // ureidopropionase, beta // 10 C1|10 // 103149 /// NM_13399	Upb1	1.25E -03	-1.19	1.72 E-02	-1.13
172072 71	---		1.25E -03	1.43	3.82 E-03	1.37
173755 26	NM_019788 // Pldn // pallidin // 2 E5|2 67.6 cM // 18457 /// ENSMUST00000005954 // Pldn	Pldn	1.25E -03	1.09	7.64 E-03	1.07
175057 42	---		1.25E -03	-1.46	4.41 E-02	-1.24
174154 30	---		1.25E -03	-1.26	2.15 E-03	-1.24
175201 67	---		1.25E -03	1.57	7.84 E-03	1.43
175295 55	---		1.25E -03	1.57	7.84 E-03	1.43
173002 77	---		1.27E -03	1.45	1.14 E-03	1.45
174940 91	NM_020289 // Olfr544 // olfactory receptor 544 // 7 E3|7 // 257926 /// ENSMUST000000512	Olfr54 4	1.27E -03	-1.13	2.46 E-03	-1.12
172299 67	ENSMUST00000073663 // Olfr1408 // olfactory receptor 1408 // 1 H3|1 // 258759 /// NM_14	Olfr14 08	1.27E -03	-1.20	3.20 E-02	-1.12
172911 46	NM_013483 // Btn1a1 // butyrophilin, subfamily 1, member A1 // 13 A3.1|13 12.0 cM // 12	Btn1a 1	1.28E -03	-1.12	2.02 E-04	-1.15
172128 74	NM_001039710 // Coq10b // coenzyme Q10 homolog B (S. cerevisiae) // 1 C1.2|1 // 67876 /	Coq10 b	1.30E -03	1.27	3.32 E-02	1.16
172795 34	---		1.30E -03	-1.20	2.34 E-03	-1.18

175 103 58	NM_145598 // Nxnl1 // nucleoredoxin-like 1 // 8 B3.3|8 // 234404 /// BC021911 // Nxnl1	Nxnl1	1.31E -03	-1.14	5.26 E-05	-1.20
174 592 88	---		1.31E -03	-1.23	1.26 E-02	-1.17
172 689 09	NM_011623 // Top2a // topoisomerase (DNA) II alpha // 11 D|11 57.0 cM // 21973 /// ENSM	Top2a	1.31E -03	-1.19	2.06 E-02	-1.13
174 486 41	---		1.33E -03	-1.20	8.49 E-04	-1.21
173 351 65	---		1.34E -03	-1.17	3.27 E-05	-1.25
172 882 69	ENSMUST00000168767 // Gm10767 // predicted gene 10767 // --- // 100038538 /// NM_001177	Gm10 767	1.35E -03	1.16	1.71 E-02	1.11
172 132 46	NM_001033373 // Cdk15 // cyclin-dependent kinase 15 // 1 C1.3|1 // 271697 /// ENSMUST00	Cdk15	1.36E -03	-1.16	9.23 E-03	-1.12
173 938 05	NM_177657 // D630003M21Rik // RIKEN cDNA D630003M21 gene // 2 H1|2 // 228846 /// ENSMUS	D6300 03M2 1Rik	1.36E -03	-1.14	6.07 E-03	-1.12
175 421 33	---		1.36E -03	-1.49	5.12 E-04	-1.56
175 477 95	NM_001198998 // Vdac3 // voltage-dependent anion channel 3 // 8 A2|8 8.0 cM // 22335 //	Vdac3	1.37E -03	1.18	1.26 E-03	1.18
172 403 23	NM_144821 // AI317395 // expressed sequence AI317395 // 10 B1|10 // 215929 /// ENSMUST0	AI317 395	1.37E -03	-1.17	1.95 E-02	-1.12
174 285 45	NM_025641 // Uqcrh // ubiquinol-cytochrome c reductase hinge protein // 4|4 C7 // 66576	Uqcrh	1.37E -03	1.23	2.92 E-03	1.21
173 654 71	NM_001081271 // Calhm1 // calcium homeostasis modulator 1 // 19 C3|19 // 546729 /// ENS	Calhm 1	1.37E -03	-1.24	7.93 E-03	-1.19
172 445 22	NR_037212 // Mir3058 // microRNA 3058 // --- // 100526463	Mir30 58	1.38E -03	-1.17	3.20 E-03	-1.16
173 542 82	NM_033037 // Cdo1 // cysteine dioxygenase 1, cytosolic // 18 C|18 23.0 cM // 12583 ///	Cdo1	1.38E -03	1.24	1.48 E-03	1.24
173 167 00	NM_001102458 // Azin1 // antizyme inhibitor 1 // 15|15 C // 54375 /// ENSMUST0000011032	Azin1	1.38E -03	1.32	2.75 E-03	1.29
175 218 75	NM_020520 // Slc25a20 // solute carrier family 25 (mitochondrial carnitine/acylcarnitin	Slc25a 20	1.38E -03	1.18	2.46 E-03	1.17

17323120	NM_023317 // Nde1 // nuclear distribution gene E homolog 1 (A nidulans) // 16 A1|16 //	Nde1	1.39E-03	1.20	2.13E-03	1.19
17341703	---		1.39E-03	1.22	2.58E-03	1.20
17290997	NM_178199 // Hist1h2bl // histone cluster 1, H2bl // 13 A2-A3|13 // 319185 /// ENSMUST0	Hist1h2bl	1.39E-03	-1.32	2.36E-02	-1.20
17456434	NM_028920 // Hyal6 // hyaluronoglucosaminidase 6 // 6 A3.1|6 // 74409 /// ENSMUST000000	Hyal6	1.39E-03	-1.26	3.98E-03	-1.22
17533992	---		1.40E-03	-1.32	6.15E-03	-1.26
17347987	XR_107094 // Gm19783 // predicted gene, 19783 // --- // 100503581	Gm19783	1.42E-03	-1.26	9.65E-03	-1.20
17322312	NR_045743 // D930007P13Rik // Riken cDNA D930007P13 gene // 15 F3|15 // 432989	D930007P13Rik	1.42E-03	-1.18	2.21E-02	-1.12
17442060	NM_177242 // Pptc7 // PTC7 protein phosphatase homolog (S. cerevisiae) // 5 F|5 // 3207	Pptc7	1.43E-03	1.28	3.24E-02	1.17
17300149	---		1.43E-03	-1.12	1.07E-02	-1.09
17378232	DQ080431 // 1700003F12Rik // RIKEN cDNA 1700003F12 gene // 2 H1|2 // 75480 /// ENSMUST0	1700003F12Rik	1.47E-03	-1.16	3.46E-04	-1.19
17393766	AK019110 // 2410019O14Rik // RIKEN cDNA 2410019O14 gene // 2|2 // 78316 /// AK019110 //	2410019O14Rik	1.47E-03	-1.15	2.76E-02	-1.10
17361605	NM_009985 // Ctsw // cathepsin W // 19 A|19 // 13041 /// ENSMUST00000025844 // Ctsw //	Ctsw	1.47E-03	-1.14	1.97E-03	-1.14
17388252	NM_001033451 // Zfp408 // zinc finger protein 408 // 2 E1|2 // 381410 /// ENSMUST000001	Zfp408	1.49E-03	1.23	3.30E-02	1.14
17275830	---		1.49E-03	1.24	1.94E-03	1.23
17274751	NM_013709 // Sh3yl1 // Sh3 domain YSC-like 1 // 12 A2|12 // 24057 /// ENSMUST0000002099	Sh3yl1	1.49E-03	1.29	1.32E-03	1.30
17274446	---		1.50E-03	-1.28	3.08E-02	-1.17
17534265	NM_194063 // Rhox3a // reproductive homeobox 3A // X A2|X // 382209 /// ENSMUST00000115	Rhox3a	1.50E-03	-1.18	1.65E-02	-1.13

173719 76	ENSMUST00000090811 // Scrn3 // secernin 3 // 2 C3|2 // 74616 /// NM_029022 // Scrn3 //	Scrn3	1.51E-03	1.27	5.35E-03	1.23
175341 54	---		1.51E-03	1.29	9.10E-04	1.31
174487 84	---		1.51E-03	1.61	1.10E-02	1.44
172032 17	---		1.53E-03	1.51	5.32E-03	1.43
175150 27	NM_001030290 // Rdh8 // retinol dehydrogenase 8 // 9 A3|9 // 235033 /// ENSMUST00000066	Rdh8	1.53E-03	-1.16	4.95E-04	-1.18
172734 79	NM_027622 // Tex19.2 // testis expressed gene 19.2 // 11 E2|11 // 70956 /// ENSMUST0000	Tex19.2	1.53E-03	-1.19	1.31E-02	-1.14
173001 35	---		1.53E-03	-1.23	1.63E-02	-1.16
175138 54	---		1.53E-03	-1.59	3.57E-03	-1.52
172995 82	ENSMUST00000100645 // Eddm3b // epididymal protein 3B // 14 C1|14 // 219026 /// NM_2035	Eddm3b	1.54E-03	-1.21	1.05E-02	-1.16
174153 73	NM_010502 // Ifna1 // interferon alpha 1 // 4 C4|4 42.6 cM // 15962 /// ENSMUST00000094	Ifna1	1.54E-03	-1.17	1.22E-02	-1.13
175201 62	NM_009742 // Bcl2a1a // B cell leukemia/lymphoma 2 related protein A1a // 9 E3.1|9 50.0	Bcl2a1a	1.54E-03	-1.23	1.26E-03	-1.23
174899 54	XR_140812 // LOC546957 // protein kinase C-binding protein 1-like // 7 B2|7 // 546957	LOC546957	1.55E-03	-1.24	6.50E-04	-1.26
173566 85	ENSMUST00000113526 // Gpha2 // glycoprotein hormone alpha 2 // 19 A|19 // 170458 /// NM	Gpha2	1.56E-03	-1.14	4.95E-04	-1.16
172168 09	NM_178690 // Rab3gap1 // RAB3 GTPase activating protein subunit 1 // 1 E3-E4|1 // 22640	Rab3gap1	1.56E-03	1.15	2.10E-04	1.18
172846 17	---		1.56E-03	-1.20	4.62E-03	-1.18
173586 58	---		1.57E-03	-1.31	2.24E-02	-1.20
172216 86	NM_001160139 // Kcnq5 // potassium voltage-gated channel, subfamily Q, member 5 // 1|1	Kcnq5	1.57E-03	1.15	1.79E-02	1.10

17285855	NM_010377 // Hist1h1t // histone cluster 1, H1t // 13|13 A3.2 // 107970 /// ENSMUST0000	Hist1h1t	1.57E-03	-1.31	9.79E-03	-1.24
17349162	NM_153515 // Ammecr1l // AMME chromosomal region gene 1-like // 18 B1|18 // 225339 ///	Ammecr1l	1.57E-03	1.17	4.37E-02	1.10
17411304	NR_028095 // Rabggtb // RAB geranylgeranyl transferase, b subunit // 3 H3|3 76.5 cM //	Rabggtb	1.58E-03	1.26	5.93E-04	1.29
17248988	NM_146469 // Olfr1381 // olfactory receptor 1381 // 11 B1.2|11 // 258461 /// ENSMUST000	Olfr1381	1.58E-03	-1.16	2.72E-03	-1.15
17517365	---		1.59E-03	-1.26	3.72E-02	-1.15
17548719	XR_140943 // LOC100861732 // uncharacterized LOC100861732 // --- // 100861732	LOC100861732	1.59E-03	-1.13	2.55E-03	-1.12
17411727	NM_013526 // Gdf6 // growth differentiation factor 6 // 4 A1|4 // 242316 /// ENSMUST000	Gdf6	1.60E-03	-1.20	9.16E-04	-1.22
17319401	NR_028282 // Snord83b // small nucleolar RNA, C/D box 83B // 15 E1|15 // 100302601	Snord83b	1.60E-03	1.48	2.58E-02	1.30
17400799	ENSMUST00000029476 // Sec22b // SEC22 vesicle trafficking protein homolog B (S. cerevis	Sec22b	1.61E-03	1.16	2.63E-03	1.15
17325014	NM_080557 // Snx4 // sorting nexin 4 // 16 B3|16 // 69150 /// ENSMUST00000023502 // Snx	Snx4	1.61E-03	1.27	1.29E-03	1.28
17310452	NM_025673 // Golph3 // golgi phosphoprotein 3 // 15 A1|15 // 66629 /// ENSMUST000000596	Golph3	1.61E-03	1.30	1.95E-02	1.20
17514515	NM_010809 // Mmp3 // matrix metallopeptidase 3 // 9 A1|9 1.0 cM // 17392 /// ENSMUST000	Mmp3	1.63E-03	-1.20	2.20E-02	-1.13
17542732	NM_181584 // Gab3 // growth factor receptor bound protein 2-associated protein 3 // X A	Gab3	1.63E-03	1.13	2.62E-06	1.24
17315255	NM_145625 // Eif4b // eukaryotic translation initiation factor 4B // 15 F3|15 // 75705	Eif4b	1.64E-03	1.16	1.68E-03	1.16
17536264	NM_177546 // Pcyt1b // phosphate cytidylyltransferase 1, choline, beta isoform // X C3|	Pcyt1b	1.64E-03	1.21	4.99E-03	1.18
17262387	---		1.64E-03	-1.14	5.82E-04	-1.15
17258642	NR_028567 // Snord1b // small nucleolar RNA, C/D box 1B // 11|11 // 100216531	Snord1b	1.64E-03	1.65	1.64E-03	1.65

173413 54	NM_134202 // Vmn1r233 // vomeronasal 1 receptor 233 // 17 A3.2|17 // 171236 /// ENSMUST	Vmn1r 233	1.64E -03	-1.32	3.80 E-02	-1.19
172100 71	---		1.65E -03	1.69	2.07 E-03	1.67
175487 30	---		1.65E -03	1.16	7.05 E-03	1.13
174179 46	NM_008190 // Guca2a // guanylate cyclase activator 2a (guanylin) // 4 D2.1|4 57.0 cM //	Guca2 a	1.65E -03	-1.14	1.43 E-04	-1.18
174860 94	NM_146913 // Olfr1348 // olfactory receptor 1348 // 7 A1|7 // 258915 /// ENSMUST0000008	Olfr13 48	1.66E -03	-1.26	7.83 E-03	-1.21
172387 58	---		1.66E -03	-1.25	1.72 E-02	-1.18
172981 39	NM_027493 // Actr8 // ARP8 actin-related protein 8 // 14 B|14 // 56249 /// ENSMUST00000	Actr8	1.68E -03	1.23	2.14 E-02	1.16
174339 42	---		1.68E -03	-1.51	9.16 E-03	-1.39
172602 95	NM_173748 // Nudcd3 // NudC domain containing 3 // 11 A1|11 // 209586 /// ENSMUST000000	Nudcd 3	1.68E -03	1.22	2.21 E-02	1.15
174942 61	NM_147120 // Olfr638 // olfactory receptor 638 // 7 E3|7 // 259124 /// ENSMUST000000981	Olfr63 8	1.68E -03	-1.29	2.70 E-02	-1.19
172890 85	NM_010049 // Dhfr // dihydrofolate reductase // 13 C3|13 43.0 cM // 13361 /// ENSMUST00	Dhfr	1.69E -03	1.16	9.81 E-04	1.17
173316 42	ENSMUST00000116584 // Mrpl39 // mitochondrial ribosomal protein L39 // 16 C3.3|16 // 27	Mrpl3 9	1.70E -03	1.23	3.18 E-03	1.21
173365 36	NM_001001177 // BC051142 // cDNA sequence BC051142 // 17 B1|17 18.0 cM // 407788 /// EN	BC051 142	1.70E -03	1.27	1.98 E-04	1.35
173415 51	---		1.71E -03	-1.20	1.61 E-04	-1.26
172843 76	L41876 // Igh-VX24 // immunoglobulin heavy chain (X24 family) // 12 F2|12 59.0 cM // 19	Igh-VX24	1.71E -03	-1.24	3.25 E-03	-1.22
173341 72	NM_001039238 // Dcpp2 // demilune cell and parotid protein 2 // 17 A3.3|17 // 630537 //	Dcpp2	1.72E -03	-1.32	1.95 E-02	-1.22
172025 71	---		1.73E -03	1.74	8.14 E-03	1.57

17362031	NM_009203 // Slc22a12 // solute carrier family 22 (organic anion/cation transporter), m	Slc22a12	1.73E-03	-1.11	1.40E-02	-1.08
17210201	---		1.74E-03	1.91	1.60E-02	1.61
17534169	NM_194063 // Rhox3a // reproductive homeobox 3A // X A2|X // 382209 /// ENSMUST00000115	Rhox3a	1.75E-03	-1.19	1.57E-02	-1.14
17448670	NM_174877 // Zar1 // zygote arrest 1 // 5 C3.2|5 // 317755 /// ENSMUST00000073528 // Za	Zar1	1.75E-03	-1.26	7.86E-04	-1.28
17455496	---		1.76E-03	-1.43	4.51E-03	-1.38
17448804	---		1.77E-03	1.87	1.03E-02	1.64
17287889	---		1.78E-03	-1.20	2.46E-04	-1.25
17293717	NM_001195435 // 1190003K10Rik // RIKEN cDNA 1190003K10 gene // 13 B3|13 // 68880	1190003K10Rik	1.78E-03	-1.25	3.50E-03	-1.23
17457874	NM_001001453 // Tas2r144 // taste receptor, type 2, member 144 // 6 B2.1|6 // 387515 //	Tas2r144	1.78E-03	-1.28	5.75E-03	-1.24
17382378	AK085201 // D530008I23 // uncharacterized LOC329358 // 2 A3|2 // 329358	D530008I23	1.78E-03	-1.16	4.19E-03	-1.14
17548616	NM_010888 // Ndufs6 // NADH dehydrogenase (ubiquinone) Fe-S protein 6 // 13 D2|13 // 40	Ndufs6	1.79E-03	1.39	1.89E-02	1.27
17493292	NM_025460 // Tmem126a // transmembrane protein 126A // 7|7 E1 // 66271 /// ENSMUST00000	Tmem126a	1.81E-03	1.28	7.01E-03	1.23
17311968	NM_009375 // Tg // thyroglobulin // 15 D3-E|15 36.4 cM // 21819 /// ENSMUST00000065916	Tg	1.81E-03	-1.19	3.69E-02	-1.11
17494273	NM_146329 // Olfr642 // olfactory receptor 642 // 7 E3|7 // 258326 /// ENSMUST000000526	Olfr642	1.82E-03	-1.22	3.52E-02	-1.14
17505309	NR_029553 // Mir140 // microRNA 140 // 8|8 // 387158	Mir140	1.82E-03	1.36	1.51E-03	1.37
17294318	---		1.83E-03	-1.16	4.18E-02	-1.10
17320073	NM_001256072 // 7530416G11Rik // RIKEN cDNA 7530416G11 gene // 15 E2|15 // 328577 /// N	7530416G11Rik	1.84E-03	-1.16	3.11E-04	-1.20

174024 00	XM_980593 // Gm9372 // predicted gene 9372 // 3 G1|3 // 668809 /// ENSMUST00000168454 /	Gm9372	1.85E-03	-1.17	1.49E-03	-1.17
174782 71	---		1.85E-03	-1.25	5.70E-03	-1.21
174128 50	ENSMUST00000040008 // Ube2r2 // ubiquitin-conjugating enzyme E2R 2 // 4|4 B1 // 67615 /	Ube2r2	1.87E-03	-1.22	7.39E-03	-1.18
175505 76	---		1.87E-03	-3.34	5.80E-03	-2.85
173721 85	NM_153405 // Rbm45 // RNA binding motif protein 45 // 2 C3|2 // 241490 /// ENSMUST00000	Rbm45	1.88E-03	1.34	2.63E-02	1.22
173069 18	NM_001040072 // Nynrin // NYN domain and retroviral integrase containing // 14 C3|14 //	Nynrin	1.88E-03	-1.24	1.22E-03	-1.25
174369 27	NM_175249 // Psapl1 // prosaposin-like 1 // 5 B3|5 // 76943 /// ENSMUST00000052224 // P	Psapl1	1.88E-03	-1.21	1.14E-02	-1.16
175488 25	XM_908648 // Gm378 // predicted pseudogene 378 // X D|X // 213454	Gm378	1.88E-03	-1.14	1.38E-02	-1.11
173864 48	---		1.89E-03	-1.22	1.45E-02	-1.16
175091 71	ENSMUST00000034049 // Slc25a4 // solute carrier family 25 (mitochondrial carrier, adeni	Slc25a4	1.89E-03	1.15	8.11E-03	1.12
174008 03	---		1.90E-03	1.29	5.33E-03	1.25
175085 12	---		1.90E-03	-1.25	1.31E-03	-1.27
172333 19	NR_038015 // Gm19395 // predicted gene, 19395 // --- // 100502820 /// BC048600 // Gm193	Gm19395	1.91E-03	1.34	2.24E-02	1.23
172857 25	NM_001162929 // Pom121l2 // POM121 membrane glycoprotein-like 2 (rat) // 13 A3.1|13 //	Pom121l2	1.91E-03	-1.16	5.18E-03	-1.14
174075 36	NM_177250 // Lingo4 // leucine rich repeat and Ig domain containing 4 // 3 F2.1|3 // 32	Lingo4	1.91E-03	-1.18	4.66E-02	-1.10
175300 00	NM_029094 // Pik3cb // phosphatidylinositol 3-kinase, catalytic, beta polypeptide // 9|	Pik3cb	1.92E-03	1.17	2.85E-03	1.16
172295 96	NR_027666 // Fcrla // Fc receptor-like A // 1 H3|1 // 98752 /// NM_001160215 // Fcrla /	Fcrla	1.93E-03	-1.14	3.59E-02	-1.09

17335364	NM_001168514 // Mapk14 // mitogen-activated protein kinase 14 // 17 A3.3|17 13.5 cM //	Mapk14	1.93E-03	1.18	1.16E-02	1.14
17225283	XM_003085323 // Gm19582 // predicted gene, 19582 // --- // 100503175	Gm19582	1.93E-03	-1.19	3.59E-04	-1.23
17549200	---		1.93E-03	-1.31	6.97E-03	-1.25
17458561	NM_007624 // Cbx3 // chromobox 3 // 6 B-C|6 26.0 cM // 12417 /// ENSMUST00000156463 //	Cbx3	1.93E-03	-1.22	3.43E-04	-1.27
17251220	---		1.94E-03	-1.20	1.18E-03	-1.21
17324745	NM_001033539 // Bex6 // brain expressed gene 6 // 16 B2|16 // 328660 /// NM_001033539 /	Bex6	1.94E-03	-1.25	2.77E-02	-1.16
17394724	---		1.95E-03	1.59	5.03E-03	1.51
17460252	---		1.95E-03	-1.31	1.45E-03	-1.32
17310922	NM_016762 // Matn2 // matrilin 2 // 15|15 B3.3 // 17181 /// ENSMUST00000022947 // Matn2	Matn2	1.96E-03	1.20	1.61E-04	1.26
17210657	---		1.96E-03	-1.23	2.54E-02	-1.15
17227086	XR_106094 // Gm19497 // predicted gene, 19497 // --- // 100502999	Gm19497	1.97E-03	-1.20	4.54E-04	-1.24
17527963	NM_028748 // Paqr5 // progestin and adipoQ receptor family member V // 9|9 C // 74090 /	Paqr5	1.97E-03	1.25	3.97E-02	1.15
17249425	ENSMUST00000109072 // Skp1a // S-phase kinase-associated protein 1A // 11 B1.3|11 31.0	Skp1a	1.97E-03	1.15	1.84E-03	1.15
17291012	NM_178184 // Hist1h2an // histone cluster 1, H2an // 13 A3.1|13 // 319170 /// ENSMUST00	Hist1h2an	1.98E-03	-1.40	4.67E-04	-1.48
17428858	---		1.98E-03	1.59	1.49E-02	1.42
17469360	NM_001081157 // Lmod3 // leiomodin 3 (fetal) // 6 D3|6 // 320502 /// ENSMUST00000095655	Lmod3	1.98E-03	-1.14	1.15E-02	-1.11
17396479	NM_031197 // Slc2a2 // solute carrier family 2 (facilitated glucose transporter), membe	Slc2a2	1.98E-03	-1.15	2.74E-03	-1.15

17207671	---		1.99E-03	1.51	7.35E-03	1.42
17367367	NM_029619 // Msrb2 // methionine sulfoxide reductase B2 // 2|2 A2 // 76467 /// ENSMUST0	Msrb2	1.99E-03	1.13	4.21E-03	1.12
17550572	---		1.99E-03	-3.76	4.72E-03	-3.28
17450675	NR_045871 // 4930428O21Rik // RIKEN cDNA 4930428O21 gene // 5 F|5 // 654809	4930428O21Rik	1.99E-03	-1.21	1.99E-02	-1.15
17446832	NM_021290 // Ucn // urocortin // 5 B1|5 18.0 cM // 22226 /// ENSMUST00000043475 // Ucn	Ucn	1.99E-03	-1.12	2.53E-02	-1.08
17209765	---		2.00E-03	2.45	2.32E-02	1.87
17394940	---		2.00E-03	-1.18	1.75E-03	-1.18
17417478	---		2.01E-03	-1.60	3.84E-04	-1.75
17206213	---		2.01E-03	1.77	9.90E-03	1.59
17323068	---		2.03E-03	1.31	1.10E-02	1.24
17415236	---		2.04E-03	-1.15	3.72E-02	-1.10
17544156	---		2.04E-03	-2.05	1.08E-02	-1.77
17289824	NM_172593 // Mier3 // mesoderm induction early response 1, family member 3 // 13 D2.2|1	Mier3	2.04E-03	1.20	8.58E-03	1.17
17204909	---		2.04E-03	-1.32	4.05E-02	-1.19
17484610	NM_001099742 // Scgb1c1 // secretoglobin, family 1C, member 1 // 7 F5|7 // 338417 /// E	Scgb1c1	2.04E-03	-1.21	4.55E-02	-1.12
17457775	---		2.05E-03	-1.25	1.60E-03	-1.26
17530906	---		2.06E-03	-1.19	6.69E-03	-1.16

174 501 17	---		2.06E -03	-1.17	2.15 E-03	-1.17
174 126 45	NM_001007589 // Akirin2 // akirin 2 // 4 A5|4 // 433693 /// ENSMUST00000084299 // Akiri	Akirin 2	2.07E -03	1.16	3.02 E-02	1.11
172 009 99	---		2.07E -03	1.37	7.58 E-03	1.30
173 055 42	NM_029131 // 4930503E14Rik // RIKEN cDNA 4930503E14 gene // 14 C1|14 // 74954 /// ENSMU	49305 03E14 Rik	2.08E -03	-1.26	1.48 E-02	-1.20
173 013 40	---		2.08E -03	-2.09	2.62 E-02	-1.66
174 056 67	ENSMUST00000029414 // Ssr3 // signal sequence receptor, gamma // 3 E1|3 // 67437 /// NM	Ssr3	2.08E -03	1.28	8.34 E-03	1.23
175 309 30	NM_001042779 // Sema3b // sema domain, immunoglobulin domain (Ig), short basic domain,	Sema3 b	2.09E -03	-1.17	1.34 E-03	-1.18
175 158 35	NM_019770 // Tmed2 // transmembrane emp24 domain trafficking protein 2 // 5 F|5 // 5633	Tmed 2	2.09E -03	1.23	1.80 E-03	1.23
175 266 14	NM_008775 // Pafah1b2 // platelet-activating factor acetylhydrolase, isoform 1b, subuni	Pafah 1b2	2.09E -03	1.15	4.60 E-03	1.13
174 854 49	ENSMUST00000033388 // Oraov1 // oral cancer overexpressed 1 // 7 F5|7 // 72284 /// NM_0	Oraov 1	2.09E -03	1.13	4.82 E-02	1.08
173 766 85	NM_001145830 // Plcb1 // phospholipase C, beta 1 // 2 F3|2 76.7 cM // 18795 /// NM_0196	Plcb1	2.09E -03	1.17	1.00 E-04	1.23
173 616 56	NM_019935 // Ovol1 // OVO homolog-like 1 (Drosophila) // 19 A|19 // 18426 /// ENSMUST00	Ovol1	2.10E -03	-1.14	3.07 E-03	-1.13
174 311 66	NM_145833 // Lin28a // lin-28 homolog A (C. elegans) // 4 D3|4 // 83557 /// ENSMUST0000	Lin28a	2.11E -03	-1.13	1.06 E-02	-1.10
172 451 52	NM_001003950 // Rab3ip // RAB3A interacting protein // 10 D2|10 62.0 cM // 216363 /// E	Rab3i p	2.11E -03	1.16	8.39 E-03	1.13
172 070 11	---		2.11E -03	1.53	3.19 E-02	1.33
173 649 17	NM_001081257 // Hpse2 // heparanase 2 // 19 C3|19 // 545291 /// ENSMUST00000099428 // H	Hpse2	2.11E -03	-1.16	6.36 E-03	-1.14
173 821 56	NM_001077190 // Abi1 // abl-interactor 1 // 2 A3|2 15.0 cM // 11308 /// NM_145994 // Ab	Abi1	2.12E -03	1.31	1.20 E-02	1.24

175106 24	---		2.13E -03	-1.31	5.38 E-03	-1.27
175176 05	NM_133976 // Imp3 // IMP3, U3 small nucleolar ribonucleoprotein, homolog (yeast) // 9 B	Imp3	2.14E -03	1.36	4.67 E-02	1.20
173754 09	NM_025777 // Duoxa2 // dual oxidase maturation factor 2 // 2 E5|2 // 66811 /// ENSMUST0	Duoxa 2	2.15E -03	-1.17	5.39 E-03	-1.15
172452 02	NM_026570 // Yeats4 // YEATS domain containing 4 // 10 D2|10 // 64050 /// ENSMUST000000	Yeats4	2.15E -03	1.16	1.06 E-02	1.13
174736 60	NM_146179 // Zfp418 // zinc finger protein 418 // 7 A1|7 // 232854 /// ENSMUST000000514	Zfp41 8	2.15E -03	1.33	2.49 E-02	1.22
175194 81	ENSMUST00000076889 // Gnb5 // guanine nucleotide binding protein (G protein), beta 5 //	Gnb5	2.16E -03	1.23	2.41 E-02	1.15
172845 51	---		2.17E -03	-1.29	3.82 E-02	-1.18
172755 76	NM_001037756 // Brms1l // breast cancer metastasis-suppressor 1-like // 12 C1|12 23.0 c	Brms1 l	2.17E -03	1.30	4.80 E-04	1.36
173337 05	NR_029536 // Mir99b // microRNA 99b // 17|17 // 387230	Mir99 b	2.17E -03	-1.27	1.74 E-03	-1.28
174577 19	---		2.18E -03	-1.21	2.98 E-04	-1.27
174593 83	---		2.19E -03	-2.06	7.93 E-03	-1.84
172781 80	NM_001195726 // Fam181a // family with sequence similarity 181, member A // 12 E|12 //	Fam1 81a	2.20E -03	-1.16	1.02 E-04	-1.22
174481 70	---		2.21E -03	-1.24	5.58 E-03	-1.21
173158 91	NM_008372 // Il7r // interleukin 7 receptor // 15 A1|15 4.6 cM // 16197 /// ENSMUST0000	Il7r	2.21E -03	1.29	7.06 E-03	1.25
172202 55	NM_030131 // Cnih4 // cornichon homolog 4 (Drosophila) // 1 H4|1 // 98417 /// ENSMUST00	Cnih4	2.22E -03	1.27	1.30 E-02	1.21
173225 78	BC145794 // 4930562C15Rik // RIKEN cDNA 4930562C15 gene // 16 A1|16 2.8 cM // 78809 ///	49305 62C15 Rik	2.22E -03	-1.14	1.58 E-03	-1.14
173890 48	XR_107384 // Gm20279 // predicted gene, 20279 // --- // 100504545	Gm20 279	2.22E -03	-1.15	3.29 E-02	-1.09

174 644 85	---		2.23E -03	-1.25	4.81 E-02	-1.14
174 686 78	NM_133934 // Isy1 // ISY1 splicing factor homolog (S. cerevisiae) // 6|6 D2 // 57905 //	Isy1	2.23E -03	1.15	3.61 E-03	1.14
172 033 97	---		2.23E -03	2.14	2.74 E-02	1.69
172 745 56	---		2.24E -03	-1.30	1.45 E-02	-1.22
175 175 76	ENSMUST00000034879 // Hmg20a // high mobility group 20A // 9|9 C // 66867 /// NM_025812	Hmg2 0a	2.24E -03	1.28	6.90 E-03	1.24
173 416 79	---		2.24E -03	-1.18	3.94 E-03	-1.17
174 407 28	NM_001012726 // Aym1 // activator of yeast meiotic promoters 1 // 5 F|5 65.0 cM // 5036	Aym1	2.25E -03	-1.20	1.67 E-03	-1.21
174 757 25	NM_001024726 // Zfp607 // zinc finger proten 607 // 7 A3|7 // 545938 /// ENSMUST0000005	Zfp60 7	2.25E -03	-1.21	2.30 E-02	-1.15
173 253 73	NM_134109 // Ildr1 // immunoglobulin-like domain containing receptor 1 // 16 B3|16 // 1	Ildr1	2.25E -03	-1.13	3.12 E-03	-1.13
172 692 02	---		2.26E -03	-1.19	7.71 E-03	-1.16
174 500 91	NR_034038 // 5430416N02Rik // RIKEN cDNA 5430416N02 gene // 5 E4|5 // 100503199	54304 16N02 Rik	2.27E -03	1.38	1.63 E-03	1.40
174 033 01	NM_145546 // Gtf2b // general transcription factor IIB // 3 H1|3 // 229906 /// ENSMUST0	Gtf2b	2.27E -03	1.24	1.60 E-04	1.32
174 944 51	NM_207146 // Olfr670 // olfactory receptor 670 // 7 E3|7 // 384703 /// ENSMUST000000504	Olfr67 0	2.28E -03	-1.18	2.91 E-02	-1.12
172 268 70	---		2.29E -03	-1.11	5.55 E-03	-1.10
175 469 82	XM_003086803 // LOC100040196 // Y-linked testis-specific protein 1-like // Y|Y // 10004	LOC10 00401 96	2.29E -03	-1.44	1.84 E-02	-1.31
173 681 22	NM_033145 // Lcn8 // lipocalin 8 // 2 A3|2 // 78076 /// ENSMUST00000038482 // Lcn8 // l	Lcn8	2.29E -03	-1.17	6.33 E-04	-1.20
175 148 41	NM_133214 // BC017612 // cDNA sequence BC017612 // 9 A2|9 // 170748 /// ENSMUST00000045	BC017 612	2.30E -03	-1.21	3.79 E-02	-1.13

17292681	---		2.31E-03	-1.14	2.24E-03	-1.14
17474633	ENSMUST00000011407 // Exoc3l2 // exocyst complex component 3-like 2 // 7 A3|7 // 74463	Exoc3l2	2.31E-03	-1.19	2.26E-03	-1.19
17455234	ENSMUST00000161859 // Rnf6 // ring finger protein (C3H2C3 type) 6 // 5 G3|5 // 74132 //	Rnf6	2.31E-03	1.12	7.27E-04	1.14
17467444	NR_004434 // Rprl1 // ribonuclease P RNA-like 1 // 6|6 // 19783	Rprl1	2.31E-03	-1.31	2.20E-03	-1.31
17282148	ENSMUST00000055262 // Vti1b // vesicle transport through interaction with t-SNAREs 1B h	Vti1b	2.32E-03	1.21	3.14E-04	1.26
17409461	NM_025723 // 4921515J06Rik // RIKEN cDNA 4921515J06 gene // 3|3 G1 // 66715 /// ENSMUST	4921515J06Rik	2.32E-03	-1.20	5.34E-04	-1.24
17326747	AK018881 // 1700066C05Rik // RIKEN cDNA 1700066C05 gene // 16 C3.3|16 // 78654 /// XM_0	1700066C05Rik	2.33E-03	-1.30	6.62E-03	-1.25
17206275	---		2.34E-03	1.42	8.45E-04	1.48
17295212	NM_012048 // Polk // polymerase (DNA directed), kappa // 13 D1|13 // 27015 /// ENSMUST0	Polk	2.36E-03	1.27	1.20E-03	1.29
17451957	ENSMUST00000031594 // Sdsl // serine dehydratase-like // 5 F|5 // 257635 /// NM_133902	Sdsl	2.36E-03	-1.17	2.89E-04	-1.21
17354793	NR_029601 // Mir143 // microRNA 143 // 18|18 // 387161	Mir143	2.36E-03	-1.18	1.18E-02	-1.14
17250624	NM_178618 // Fam83g // family with sequence similarity 83, member G // 11 B2|11 // 6964	Fam83g	2.37E-03	-1.15	1.24E-03	-1.16
17428457	NM_007822 // Cyp4a14 // cytochrome P450, family 4, subfamily a, polypeptide 14 // 4 D1|	Cyp4a14	2.38E-03	-1.26	1.44E-02	-1.20
17225785	NM_001105667 // Dtymk // deoxythymidylate kinase // 1 D|1 38.0 cM // 21915 /// ENSMUST0	Dtymk	2.38E-03	1.21	3.15E-02	1.14
17202247	---		2.39E-03	1.77	1.60E-03	1.81
17476877	---		2.40E-03	-1.63	5.08E-04	-1.78
17275436	NM_198111 // Akap6 // A kinase (PRKA) anchor protein 6 // 12 C1|12 // 238161 /// ENSMUS	Akap6	2.40E-03	1.18	4.43E-03	1.17

174133 99	---		2.41E -03	-1.11	4.97 E-04	-1.13
172814 53	---		2.42E -03	-1.33	3.29 E-04	-1.42
172514 30	NM_009080 // Rpl26 // ribosomal protein L26 // 11 B3|11 // 19941 /// ENSMUST00000167436	Rpl26	2.42E -03	-1.19	1.70 E-03	-1.20
175437 53	NM_027591 // Dmrtc1a // DMRT-like family C1a // X D|X // 70887 /// ENSMUST00000033686 /	Dmrtc 1a	2.42E -03	-1.09	4.28 E-03	-1.09
173524 12	NM_026157 // Mtpap // mitochondrial poly(A) polymerase // 18 A1|18 // 67440 /// ENSMUST	Mtpap	2.42E -03	-1.22	3.01 E-02	-1.14
172973 85	---		2.42E -03	-1.26	3.95 E-02	-1.16
175288 50	NM_016963 // Tmod3 // tropomodulin 3 // 9 D|9 38.0 cM // 50875 /// ENSMUST00000072232 /	Tmod 3	2.43E -03	1.18	1.06 E-02	1.15
174268 08	NM_177863 // Frem1 // Fras1 related extracellular matrix protein 1 // 4 C3|4 // 329872	Frem1	2.44E -03	-1.15	1.13 E-02	-1.12
172209 37	---		2.45E -03	-1.17	3.26 E-02	-1.11
173586 11	---		2.47E -03	-1.19	1.52 E-03	-1.20
172814 04	NR_045049 // Gm20063 // predicted gene, 20063 // 12 C1 // 100504101	Gm20 063	2.47E -03	1.30	1.14 E-02	1.24
173050 12	NM_183209 // Gprin2 // G protein regulated inducer of neurite outgrowth 2 // 14 B|14 //	Gprin 2	2.47E -03	-1.15	2.64 E-03	-1.14
175340 95	NM_016783 // Pgrmc1 // progesterone receptor membrane component 1 // X A3.3|X // 53328	Pgrmc 1	2.48E -03	1.19	8.26 E-03	1.16
173758 80	NM_009633 // Adra2b // adrenergic receptor, alpha 2b // 2 F1|2 71.0 cM // 11552 /// ENS	Adra2 b	2.51E -03	-1.17	3.71 E-04	-1.22
172349 17	NM_008504 // Gzmm // granzyme M (lymphocyte met-ase 1) // 10 C|10 43.0 cM // 16904 ///	Gzmm	2.52E -03	-1.13	7.52 E-05	-1.19
175127 52	ENSMUST00000077208 // LOC100046297 // 40S ribosomal protein S26-like // 15|15 // 100046	LOC10 00462 97	2.52E -03	1.39	1.30 E-02	1.30
172033 67	---		2.53E -03	1.31	1.70 E-02	1.23

173854 86	NM_028118 // Wdsub1 // WD repeat, SAM and U-box domain containing 1 // 2|2 C3 // 72137	Wdsub1	2.53E-03	1.16	1.17E-02	1.12
173924 14	NM_172859 // 6330439K17Rik // RIKEN cDNA 6330439K17 gene // 2 G1|2 // 241688 /// ENSMUS	6330439K17Rik	2.53E-03	1.16	3.77E-02	1.10
173383 00	NR_045945 // Gm14872 // predicted gene 14872 // --- // 100415787 /// ENSMUST00000113299	Gm14872	2.53E-03	-1.19	1.33E-03	-1.21
174854 24	---		2.53E-03	-1.14	1.66E-03	-1.15
175041 38	NM_011332 // Ccl17 // chemokine (C-C motif) ligand 17 // 8 C5|8 45.0 cM // 20295 /// EN	Ccl17	2.54E-03	1.36	2.10E-02	1.25
175478 87	XR_141278 // LOC100861922 // uncharacterized LOC100861922 // --- // 100861922	LOC100861922	2.54E-03	-1.15	1.17E-03	-1.17
174074 81	NM_028628 // Lce1l // late cornified envelope 1L // 3 F1|3 // 73730 /// ENSMUST00000054	Lce1l	2.55E-03	-1.22	1.10E-02	-1.17
175491 78	---		2.56E-03	-1.17	6.66E-03	-1.14
174128 53	---		2.56E-03	-1.38	4.46E-03	-1.35
174735 23	XM_912340 // Gm5321 // predicted gene 5321 // 7 A1|7 // 384525 /// ENSMUST00000072050 /	Gm5321	2.57E-03	-1.30	9.28E-04	-1.35
174110 14	NR_033542 // Gm10636 // predicted gene 10636 // --- // 100038607	Gm10636	2.58E-03	-1.26	1.22E-02	-1.21
174981 22	NR_002452 // Nctc1 // non-coding transcript 1 // 7 F5|7 69.0 cM // 330677	Nctc1	2.59E-03	-1.14	1.62E-02	-1.11
174392 80	NM_001200055 // AU018829 // expressed sequence AU018829 // 5 E3|5 // 100041253 /// NM_0	AU018829	2.59E-03	-1.18	3.20E-02	-1.12
174556 77	NM_001200055 // AU018829 // expressed sequence AU018829 // 5 E3|5 // 100041253 /// NM_0	AU018829	2.59E-03	-1.18	3.20E-02	-1.12
174556 86	NM_001200055 // AU018829 // expressed sequence AU018829 // 5 E3|5 // 100041253 /// NM_0	AU018829	2.59E-03	-1.18	3.20E-02	-1.12
174557 31	NM_001200055 // AU018829 // expressed sequence AU018829 // 5 E3|5 // 100041253 /// NM_0	AU018829	2.59E-03	-1.18	3.20E-02	-1.12
172207 57	---		2.60E-03	1.44	5.73E-04	1.54

174370 54	ENSMUST00000114126 // Stx18 // syntaxin 18 // 5 B3|5 // 71116 /// NM_026959 // Stx18 //	Stx18	2.60E -03	1.14	8.02 E-03	1.12
174672 39	---		2.60E -03	-1.32	2.66 E-02	-1.21
173982 72	NM_027843 // Arl14 // ADP-ribosylation factor-like 14 // 3|3 E3 // 71619 /// BC104368 /	Arl14	2.60E -03	-1.23	9.38 E-03	-1.19
173382 80	NM_020048 // Med20 // mediator complex subunit 20 // 17 C|17 // 56771 /// ENSMUST000000	Med20	2.61E -03	1.15	4.27 E-03	1.14
172183 61	---		2.61E -03	1.30	3.70 E-02	1.19
175135 81	BC147528 // 1700018B08Rik // RIKEN cDNA 1700018B08 gene // 8 E1|8 // 76405 /// BC147542	1700018B08 Rik	2.63E -03	-1.13	4.16 E-02	-1.08
173981 52	ENSMUST00000077271 // Gfm1 // G elongation factor, mitochondrial 1 // 3 E1|3 31.6 cM //	Gfm1	2.63E -03	1.18	8.04 E-03	1.15
173435 25	---		2.64E -03	-1.20	5.49 E-03	-1.18
173002 09	---		2.64E -03	-1.18	2.38 E-02	-1.12
174093 86	NM_029522 // Gpsm2 // G-protein signalling modulator 2 (AGS3-like, C. elegans) // 3 F3|	Gpsm2	2.65E -03	1.09	1.43 E-04	1.12
172943 77	ENSMUST00000022062 // Sdha // succinate dehydrogenase complex, subunit A, flavoprotein	Sdha	2.66E -03	1.20	2.18 E-02	1.15
174411 22	NR_036616 // Srsf9 // serine/arginine-rich splicing factor 9 // 5 F|5 // 108014 /// NM_	Srsf9	2.66E -03	1.20	2.62 E-03	1.20
173741 35	---		2.67E -03	-1.43	4.98 E-04	-1.53
173776 21	NM_001160363 // 5430405G05Rik // RIKEN cDNA 5430405G05 gene // 2 G3|2 // 108832 /// ENS	5430405G05 Rik	2.67E -03	-1.14	1.64 E-03	-1.14
172909 99	NM_178211 // Hist1h4k // histone cluster 1, H4k // 13 A2-A3|13 // 319160 /// BC096767 /	Hist1h4k	2.67E -03	-1.20	6.69 E-03	-1.17
173309 43	---		2.68E -03	-1.37	1.86 E-02	-1.27
174621 91	NM_001166430 // Hnrnpf // heterogeneous nuclear ribonucleoprotein F // 6 F1|6 // 98758	Hnrnpf	2.69E -03	1.15	2.54 E-02	1.10

17214753	NM_010570 // Irs1 // insulin receptor substrate 1 // 1 C5|1 57.0 cM // 16367 /// ENSMUS	Irs1	2.70E-03	-1.15	4.32E-03	-1.14
17408099	ENSMUST00000154679 // Polr3c // polymerase (RNA) III (DNA directed) polypeptide C // 3|	Polr3c	2.70E-03	1.16	8.22E-03	1.14
17257556	---		2.71E-03	1.43	1.89E-02	1.31
17487590	NM_001201364 // Gm10662 // predicted gene 10662 // 7 A3|7 // 100043665 /// NM_001034904	Gm10662	2.71E-03	-1.20	7.81E-04	-1.23
17487651	NM_001201364 // Gm10662 // predicted gene 10662 // 7 A3|7 // 100043665 /// NM_001034904	Gm10662	2.71E-03	-1.20	7.81E-04	-1.23
17405673	---		2.71E-03	1.37	9.54E-03	1.30
17213428	ENSMUST00000060608 // Cyp20a1 // cytochrome P450, family 20, subfamily a, polypeptide 1	Cyp20a1	2.72E-03	1.38	2.04E-02	1.27
17202407	---		2.72E-03	-1.37	4.03E-03	-1.35
17286796	NM_177808 // Gm5082 // predicted gene 5082 // 13 A4|13 // 328231 /// NM_001145878 // Gm	Gm5082	2.73E-03	-1.18	1.57E-02	-1.14
17545399	NM_001164578 // Tsr2 // TSR2, 20S rRNA accumulation, homolog (S. cerevisiae) // X F3|X	Tsr2	2.73E-03	1.42	2.09E-02	1.29
17426251	NM_010616 // Kif12 // kinesin family member 12 // 4|4 C1 // 16552 /// ENSMUST0000003004	Kif12	2.73E-03	-1.14	1.99E-02	-1.11
17344682	---		2.74E-03	-1.16	2.91E-02	-1.11
17247921	NM_027260 // Vrk2 // vaccinia related kinase 2 // 11 A3.3|11 // 69922 /// NM_001252447	Vrk2	2.74E-03	-1.12	3.27E-03	-1.12
17218965	NM_173029 // Adcy10 // adenylate cyclase 10 // 1 H2.3|1 // 271639 /// ENSMUST0000002785	Adcy10	2.74E-03	-1.15	4.24E-04	-1.19
17399233	---		2.75E-03	-1.37	1.27E-02	-1.29
17483303	ENSMUST00000033095 // Prr14 // proline rich 14 // 7 F3|7 // 233895 /// NM_145589 // Prr	Prr14	2.75E-03	1.08	1.48E-03	1.09
17418997	---		2.75E-03	-1.14	3.37E-02	-1.09

17432587	---		2.76E-03	1.24	1.13E-03	1.27
17312183	ENSMUST00000050234 // Jrk // jerky // 15 D3|15 42.8 cM // 16469 /// NR_033197 // 493342	Jrk	2.77E-03	-1.17	1.07E-02	-1.14
17502992	---		2.77E-03	-1.23	3.07E-02	-1.16
17528934	NM_012033 // Tinag // tubulointerstitial nephritis antigen // 9 D|9 // 26944 /// ENSMUS	Tinag	2.78E-03	-1.13	1.86E-02	-1.09
17284803	NR_045947 // D230030E09Rik // Riken cDNA D230030E09 gene // --- // 100384890	D230030E09Rik	2.79E-03	-1.21	1.11E-03	-1.23
17505013	NM_025518 // Dus2l // dihydrouridine synthase 2-like (SMM1, S. cerevisiae) // 8|8 D2 //	Dus2l	2.79E-03	1.12	4.33E-02	1.08
17443112	---		2.80E-03	-1.17	3.12E-04	-1.22
17341076	NR_027986 // 4930474M22Rik // RIKEN cDNA 4930474M22 gene // 17 A2|17 // 74917 /// BC089	4930474M22Rik	2.81E-03	-1.18	4.16E-03	-1.17
17318105	AK153212 // I830127L07Rik // RIKEN cDNA I830127L07 gene // 15 D3|15 // 546643 /// XM_90	I830127L07Rik	2.82E-03	-1.26	1.30E-02	-1.20
17526803	---		2.82E-03	-1.58	1.56E-03	-1.64
17255822	NR_045496 // 2410003L11Rik // RIKEN cDNA 2410003L11 gene // 11 D|11 // 69729 /// NR_045	2410003L11Rik	2.83E-03	-1.15	3.66E-04	-1.19
17248421	---		2.83E-03	-1.18	3.44E-02	-1.12
17286882	NM_023554 // Nol7 // nucleolar protein 7 // 13 A4|13 // 70078 /// ENSMUST00000071926 //	Nol7	2.84E-03	1.24	2.44E-03	1.25
17458110	NR_024202 // Rny3 // RNA, Y3 small cytoplasmic (associated with Ro protein) // 6|6 22.0	Rny3	2.84E-03	-1.62	2.78E-02	-1.40
17262926	NM_172258 // Slc36a3 // solute carrier family 36 (proton/amino acid symporter), member	Slc36a3	2.84E-03	-1.20	5.00E-02	-1.12
17246113	NM_028051 // Slc39a5 // solute carrier family 39 (metal ion transporter), member 5 // 1	Slc39a5	2.84E-03	-1.13	2.72E-03	-1.13
17505144	NR_045470 // 1110028F18Rik // RIKEN cDNA 1110028F18 gene // 8|8 // 68692 /// NR_045470	1110028F18Rik	2.85E-03	-1.28	1.55E-02	-1.21

17232055	NM_011361 // Sgk1 // serum/glucocorticoid regulated kinase 1 // 10 A3|10 // 20393 /// E	Sgk1	2.85E-03	1.41	2.80E-03	1.41
17455668	NM_001243937 // Gm3139 // predicted gene 3139 // 5 E3|5 // 100041102 /// NM_001243938 /	Gm3139	2.86E-03	-1.18	1.24E-02	-1.14
17549140	---		2.86E-03	-1.11	3.13E-04	-1.14
17238679	NM_146378 // Olfr794 // olfactory receptor 794 // 10 D3|10 // 258375 /// ENSMUST0000005	Olfr794	2.87E-03	-1.19	1.12E-02	-1.16
17252609	NM_033321 // P2rx5 // purinergic receptor P2X, ligand-gated ion channel, 5 // 11|11 B5	P2rx5	2.87E-03	-1.15	4.76E-04	-1.19
17484080	NM_198017 // Fam175b // family with sequence similarity 175, member B // 7 F3|7 // 1093	Fam175b	2.87E-03	1.21	6.23E-03	1.19
17237084	ENSMUST00000020049 // Ccdc59 // coiled-coil domain containing 59 // 10 D1|10 59.0 cM //	Ccdc59	2.88E-03	1.27	1.08E-03	1.31
17477884	NM_025330 // Hsd17b14 // hydroxysteroid (17-beta) dehydrogenase 14 // 7 B4|7 // 66065 /	Hsd17b14	2.88E-03	-1.14	2.08E-02	-1.10
17550556	---		2.89E-03	-1.81	4.79E-02	-1.45
17495214	---		2.89E-03	-1.34	2.37E-02	-1.24
17424998	NM_011728 // Xpa // xeroderma pigmentosum, complementation group A // 4 C2|4 21.5 cM //	Xpa	2.89E-03	1.23	7.67E-04	1.27
17360444	NM_009348 // Tectb // tectorin beta // 19 D2|19 52.0 cM // 21684 /// ENSMUST00000025936	Tectb	2.90E-03	-1.12	4.88E-03	-1.11
17236527	NR_038014 // Gm17745 // predicted gene, 17745 // 10 C2|10 // 432488	Gm17745	2.91E-03	-1.16	7.46E-04	-1.19
17355935	ENSMUST00000025840 // Mtl5 // metallothionein-like 5, testis-specific (tesmin) // 19|19	Mtl5	2.92E-03	-1.14	2.69E-05	-1.23
17247638	---		2.93E-03	-1.10	7.36E-05	-1.15
17242232	NM_009929 // Col18a1 // collagen, type XVIII, alpha 1 // 10 B5-C1|10 41.3 cM // 12822 /	Col18a1	2.93E-03	-1.16	2.35E-04	-1.21
17288414	NM_138596 // Med10 // mediator of RNA polymerase II transcription, subunit 10 homolog (	Med10	2.94E-03	1.29	4.16E-02	1.18

174064 33	NM_001001650 // Prss48 // protease, serine, 48 // 3 F1|3 // 368202 /// ENSMUST000000613	Prss48	2.96E-03	-1.14	1.42 E-03	-1.15
173610 97	---		2.96E-03	-1.44	1.94 E-02	-1.32
172522 72	ENSMUST00000048807 // Mis12 // MIS12 homolog (yeast) // 11 B4|11 // 67139 /// NM_025993	Mis12	2.96E-03	1.29	1.75 E-02	1.22
175465 47	XM_003085466 // LOC100039675 // uncharacterized LOC100039675 // Y|Y // 100039675 /// XM	LOC10 00396 75	2.97E-03	-1.17	1.02 E-02	-1.14
175466 11	XM_003085466 // LOC100039675 // uncharacterized LOC100039675 // Y|Y // 100039675	LOC10 00396 75	2.97E-03	-1.17	1.02 E-02	-1.14
175472 00	XM_003085466 // LOC100039675 // uncharacterized LOC100039675 // Y|Y // 100039675 /// XM	LOC10 00396 75	2.97E-03	-1.17	1.02 E-02	-1.14
175472 17	XM_003085466 // LOC100039675 // uncharacterized LOC100039675 // Y|Y // 100039675 /// XM	LOC10 00396 75	2.97E-03	-1.17	1.02 E-02	-1.14
175473 24	XM_003085466 // LOC100039675 // uncharacterized LOC100039675 // Y|Y // 100039675 /// XM	LOC10 00396 75	2.97E-03	-1.17	1.02 E-02	-1.14
175473 35	XM_003085466 // LOC100039675 // uncharacterized LOC100039675 // Y|Y // 100039675 /// XM	LOC10 00396 75	2.97E-03	-1.17	1.02 E-02	-1.14
175465 53	XM_003085641 // LOC100504515 // uncharacterized LOC100504515 // --- // 100504515	LOC10 05045 15	2.97E-03	-1.17	1.02 E-02	-1.14
175472 08	XM_003085641 // LOC100504515 // uncharacterized LOC100504515 // --- // 100504515 /// XM	LOC10 05045 15	2.97E-03	-1.17	1.02 E-02	-1.14
175471 27	XM_003689069 // LOC100862076 // uncharacterized LOC100862076 // --- // 100862076 /// XM	LOC10 08620 76	2.97E-03	-1.17	1.02 E-02	-1.14
175465 32	---		2.97E-03	-1.17	1.02 E-02	-1.14
175465 35	---		2.97E-03	-1.17	1.02 E-02	-1.14
175465 44	---		2.97E-03	-1.17	1.02 E-02	-1.14
175471 07	---		2.97E-03	-1.17	1.02 E-02	-1.14
175471 38	---		2.97E-03	-1.17	1.02 E-02	-1.14

175 472 11	---		2.97E -03	-1.17	1.02 E-02	-1.14
175 473 12	---		2.97E -03	-1.17	1.02 E-02	-1.14
174 106 93	NR_040545 // 4930425O10Rik // RIKEN cDNA 4930425O10 gene // 3|3 // 74635	49304 25O10 Rik	2.97E -03	-1.19	9.48 E-03	-1.16
174 496 29	---		2.98E -03	1.44	2.21 E-02	1.31
172 086 07	---		2.98E -03	-1.30	6.79 E-03	-1.27
172 556 04	NM_001079869 // Hoxb3 // homeobox B3 // 11 D|11 56.06 cM // 15410 /// NM_010458 // Hoxb	Hoxb3	2.98E -03	-1.13	7.66 E-04	-1.16
174 176 54	---		2.99E -03	-1.47	3.88 E-03	-1.46
172 608 37	---		2.99E -03	-1.31	1.05 E-03	-1.36
172 096 37	---		3.00E -03	1.72	1.27 E-04	2.11
174 106 98	NM_001040690 // Rap1gds1 // RAP1, GTP-GDP dissociation stimulator 1 // 3 H1|3 // 229877	Rap1g ds1	3.00E -03	1.14	7.57 E-03	1.12
172 507 69	---		3.00E -03	-1.18	3.90 E-02	-1.12
173 897 04	AK044780 // A930104D05Rik // RIKEN cDNA A930104D05 gene // 2 E5|2 // 320600	A9301 04D05 Rik	3.01E -03	-1.17	1.38 E-02	-1.13
174 174 22	NM_008389 // Ipp // IAP promoted placental gene // 4 D1|4 51.4 cM // 16351 /// ENSMUST0	Ipp	3.01E -03	1.27	8.07 E-04	1.32
175 473 15	XM_003085466 // LOC100039675 // uncharacterized LOC100039675 // Y|Y // 100039675 /// XM	LOC10 00396 75	3.01E -03	-1.13	1.80 E-02	-1.10
174 805 55	NM_028145 // Klhl35 // kelch-like 35 (Drosophila) // 7|7 F1 // 72184 /// ENSMUST0000003	Klhl35	3.02E -03	-1.17	2.50 E-03	-1.17
172 430 15	---		3.04E -03	-1.28	1.39 E-05	-1.50
174 654 80	NR_029750 // Mir96 // microRNA 96 // 6|6 // 723886	Mir96	3.04E -03	-1.25	5.99 E-03	-1.22

175 480 63	BC044896 // Fam53c // family with sequence similarity 53, member C // 18 B1|18 // 66306	Fam5 3c	3.04E -03	-1.11	1.58 E-02	-1.08
172 411 57	NR_033616 // Pldi // polymorphic derived intron containing // 10 B4|10 // 73616	Pldi	3.04E -03	-1.20	3.49 E-02	-1.13
173 739 30	NM_028747 // 0610012H03Rik // RIKEN cDNA 0610012H03 gene // 2 E3|2 // 74088 /// ENSMUST	06100 12H03 Rik	3.05E -03	-1.12	1.13 E-02	-1.10
175 191 84	NM_010890 // Nedd4 // neural precursor cell expressed, developmentally down-regulated 4	Nedd4	3.05E -03	1.18	1.77 E-02	1.14
172 827 30	---		3.06E -03	-1.68	1.73 E-02	-1.49
172 353 54	XR_106535 // Gm19876 // predicted gene, 19876 // --- // 100503758	Gm19 876	3.06E -03	-1.46	1.40 E-02	-1.36
175 489 50	NM_025424 // Nenf // neuron derived neurotrophic factor // 1 H6|1 // 66208 /// ENSMUST0	Nenf	3.08E -03	-1.47	2.45 E-02	-1.33
172 062 15	---		3.08E -03	2.13	1.73 E-02	1.80
175 333 57	NR_038158 // 5730405O15Rik // RIKEN cDNA 5730405O15 gene // X|X // 70489 /// NR_038158	57304 05O15 Rik	3.08E -03	1.15	6.04 E-03	1.14
175 148 28	BC110660 // Taf1d // TATA box binding protein (Tbp)-associated factor, RNA polymerase I	Taf1d	3.09E -03	-1.49	2.61 E-02	-1.33
172 010 43	---		3.10E -03	1.42	2.06 E-03	1.45
172 341 92	ENSMUST00000020081 // Zwint // ZW10 interactor // 10 B5.3|10 38.0 cM // 52696 /// NM_02	Zwint	3.13E -03	1.10	1.84 E-02	1.07
172 001 47	---		3.15E -03	1.41	3.36 E-03	1.41
172 219 23	---		3.15E -03	-1.84	5.07 E-03	-1.78
172 275 09	AK016750 // 4933409D19Rik // RIKEN cDNA 4933409D19 gene // 1 E4-F|1 // 71084 /// XR_107	49334 09D19 Rik	3.15E -03	-1.19	1.91 E-02	-1.14
172 090 47	---		3.17E -03	1.62	1.70 E-02	1.46
172 835 46	---		3.17E -03	1.37	3.05 E-02	1.25

17236155	ENSMUST00000130320 // Fbxo7 // F-box protein 7 // 10 C1|10 // 69754 /// NM_153195 // Fb	Fbxo7	3.17E-03	1.12	5.95E-03	1.11
17332923	NM_001199948 // Dynlt1f // dynein light chain Tctex-type 1F // 17 A1|17 // 100040531 //	Dynlt1f	3.18E-03	1.14	3.17E-03	1.14
17456918	NM_025350 // Cpa1 // carboxypeptidase A1, pancreatic // 6 B1|6 6.6 cM // 109697 /// ENS	Cpa1	3.18E-03	-1.14	6.68E-04	-1.16
17402410	---		3.19E-03	-1.13	8.54E-04	-1.15
17382848	XR_107351 // Gm13553 // predicted gene 13553 // 2|2 18.42 cM // 100504023	Gm13553	3.19E-03	-1.24	2.03E-02	-1.18
17464571	NR_030250 // Mir489 // microRNA 489 // 6|6 // 723877	Mir489	3.20E-03	-1.20	3.67E-02	-1.13
17520602	---		3.20E-03	1.15	1.86E-02	1.11
17322075	NM_008475 // Krt4 // keratin 4 // 15 F3|15 58.84 cM // 16682 /// ENSMUST00000023797 //	Krt4	3.20E-03	-1.18	3.10E-03	-1.18
17478001	NM_001033170 // Fam83e // family with sequence similarity 83, member E // 7 B4|7 // 738	Fam83e	3.21E-03	-1.15	9.60E-04	-1.18
17269192	---		3.22E-03	-1.17	3.60E-03	-1.17
17506400	NM_153775 // Ctu2 // cytosolic thiouridylase subunit 2 homolog (S. pombe) // 8 E1|8 //	Ctu2	3.22E-03	-1.20	8.33E-04	-1.23
17548224	AK138039 // Gm10757 // predicted gene 10757 // --- // 100038551 /// XR_141985 // Gm1075	Gm10757	3.22E-03	-1.18	4.53E-02	-1.11
17464352	NM_027059 // 1700023A16Rik // RIKEN cDNA 1700023A16 gene // 6 G3|6 // 69371 /// ENSMUST	1700023A16Rik	3.23E-03	-1.18	6.83E-03	-1.16
17365148	---		3.23E-03	-1.19	3.02E-02	-1.13
17296723	ENSMUST00000112787 // Gm3099 // predicted gene 3099 // 14 A1|14 // 100041019	Gm3099	3.23E-03	-1.25	2.29E-03	-1.26
17352981	---		3.23E-03	1.51	2.45E-04	1.72
17377197	---		3.25E-03	1.42	2.74E-03	1.43

17200207	---		3.25E-03	1.44	3.04E-02	1.29
17266370	NR_030702 // Snord4a // small nucleolar RNA, C/D box 4A // 11|11 // 100216539 /// ENSMU	Snord4a	3.27E-03	1.55	1.05E-02	1.45
17385396	---		3.28E-03	-2.81	4.76E-02	-1.94
17455298	NR_038157 // 2210019I11Rik // RIKEN cDNA 2210019I11 gene // 5|5 // 70147	2210019I11Rik	3.29E-03	-1.17	4.66E-03	-1.16
17422384	NM_027504 // Prdm16 // PR domain containing 16 // 4 E2|4 // 70673 /// NM_001177995 // P	Prdm16	3.29E-03	-1.15	5.56E-04	-1.18
17388590	---		3.30E-03	1.43	1.04E-02	1.36
17347443	NR_033462 // Gm10494 // predicted gene 10494 // 17 E3|17 // 100038718	Gm10494	3.31E-03	-1.16	2.83E-02	-1.11
17426434	NM_019967 // Dbc1 // deleted in bladder cancer 1 (human) // 4|4 C2 // 56710 /// ENSMUST	Dbc1	3.31E-03	1.16	1.06E-02	1.13
17363799	ENSMUST00000016639 // 5033414D02Rik // RIKEN cDNA 5033414D02 gene // 19 C1|19 // 67759	5033414D02Rik	3.32E-03	1.15	9.12E-03	1.13
17200021	---		3.32E-03	1.39	6.56E-03	1.35
17291964	NM_025380 // Eef1e1 // eukaryotic translation elongation factor 1 epsilon 1 // 13|13 A5	Eef1e1	3.32E-03	1.28	4.69E-03	1.26
17206375	---		3.33E-03	1.80	1.37E-03	1.92
17405463	NM_133200 // P2ry14 // purinergic receptor P2Y, G-protein coupled, 14 // 3 D|3 29.5 cM	P2ry14	3.33E-03	1.34	1.18E-03	1.39
17512598	NM_027960 // Dpep3 // dipeptidase 3 // 8 D3|8 // 71854 /// ENSMUST00000034371 // Dpep3	Dpep3	3.34E-03	-1.10	4.53E-03	-1.09
17425010	ENSMUST00000095097 // Foxe1 // forkhead box E1 // 4 B1|4 21.5 cM // 110805	Foxe1	3.35E-03	-1.12	7.78E-04	-1.14
17496589	ENSMUST00000032912 // Qprt // quinolinate phosphoribosyltransferase // 7 F3|7 // 67375	Qprt	3.36E-03	-1.16	6.49E-03	-1.14
17286190	ENSMUST00000023602 // Prl2a1 // prolactin family 2, subfamily a, member 1 // 13 A3.1|13	Prl2a1	3.37E-03	-1.21	1.89E-02	-1.16

174245 92	---		3.38E -03	-1.21	2.54 E-02	-1.15
172075 69	---		3.38E -03	-1.22	1.27 E-02	-1.18
173168 65	---		3.39E -03	-1.27	3.41 E-04	-1.36
173010 55	NM_183175 // C1qtnf9 // C1q and tumor necrosis factor related protein 9 // 14 D1|14 //	C1qtnf 9	3.39E -03	-1.16	3.33 E-02	-1.11
173535 99	NM_011397 // Slc23a1 // solute carrier family 23 (nucleobase transporters), member 1 //	Slc23a 1	3.41E -03	-1.12	2.79 E-03	-1.12
174937 23	NM_025668 // Spcs2 // signal peptidase complex subunit 2 homolog (S. cerevisiae) // 7 E	Spcs2	3.41E -03	1.21	1.14 E-02	1.18
173793 28	ENSMUST00000018470 // Ywhab // tyrosine 3-monooxygenase/tryptophan 5-monooxygenase acti	Ywhab	3.41E -03	1.18	2.82 E-02	1.13
175326 29	---		3.41E -03	-3.46	7.30 E-03	-3.06
173000 09	---		3.43E -03	-1.16	4.55 E-03	-1.16
174675 54	---		3.45E -03	-1.47	1.25 E-03	-1.55
175253 24	---		3.45E -03	-1.17	7.40 E-03	-1.15
174768 79	---		3.46E -03	-1.33	7.39 E-04	-1.41
175146 64	---		3.47E -03	-1.28	1.66 E-04	-1.41
174976 37	ENSMUST00000051943 // Olfr524 // olfactory receptor 524 // 7 F4|7 // 258055 /// NM_0010	Olfr52 4	3.47E -03	-1.19	1.37 E-02	-1.16
172739 84	ENSMUST00000085745 // Wdr35 // WD repeat domain 35 // 12 A1.3|12 // 74682 /// NM_172470	Wdr3 5	3.47E -03	1.22	1.66 E-02	1.17
172193 97	ENSMUST00000068584 // Cd48 // CD48 antigen // 1 H3|1 93.3 cM // 12506 /// NM_007649 //	Cd48	3.48E -03	-1.21	9.95 E-03	-1.18
173468 12	XM_003085140 // Gm10495 // predicted gene 10495 // 17|17 35.3 cM // 100504611 /// ENSMU	Gm10 495	3.48E -03	1.32	7.13 E-04	1.40

172 256 03	NM_172850 // Ankmy1 // ankyrin repeat and MYND domain containing 1 // 1 D|1 // 241158 /	Ankm y1	3.48E -03	-1.12	7.65 E-03	-1.10
174 736 39	NM_021323 // Usp29 // ubiquitin specific peptidase 29 // 7 A1|7 6.5 cM // 57775 /// ENS	Usp29	3.48E -03	1.22	2.87 E-03	1.22
173 242 74	NR_027488 // Senp2 // SUMO/sentrin specific peptidase 2 // 16 B1|16 // 75826 /// NM_029	Senp2	3.49E -03	1.19	6.96 E-04	1.23
172 923 33	NM_207232 // Ptpdc1 // protein tyrosine phosphatase domain containing 1 // 13 A5|13 //	Ptpdc 1	3.51E -03	1.12	1.11 E-03	1.13
173 822 03	NM_080462 // Hnmt // histamine N-methyltransferase // 2 A3|2 // 140483 /// ENSMUST00000	Hnmt	3.51E -03	1.29	6.46 E-03	1.26
172 552 58	---		3.51E -03	-1.26	3.36 E-02	-1.17
172 114 39	---		3.51E -03	-1.51	3.72 E-02	-1.33
173 269 44	---		3.53E -03	-1.13	1.74 E-02	-1.10
174 431 48	NM_009902 // Cldn3 // claudin 3 // 5 G2|5 75.0 cM // 12739 /// ENSMUST00000094245 // Cl	Cldn3	3.54E -03	-1.13	5.73 E-06	-1.24
174 284 19	---		3.60E -03	-1.19	9.41 E-04	-1.22
173 729 00	NM_146771 // Olfr1176 // olfactory receptor 1176 // 2 D|2 // 258767 /// ENSMUST00000057	Olfr11 76	3.60E -03	-1.18	4.39 E-03	-1.18
175 259 64	---		3.61E -03	-1.34	2.03 E-02	-1.26
174 388 80	---		3.62E -03	-1.24	7.11 E-03	-1.22
172 080 05	---		3.64E -03	1.46	5.78 E-03	1.42
173 486 79	---		3.64E -03	1.41	1.96 E-02	1.30
174 730 91	ENSMUST00000111535 // Amn1 // antagonist of mitotic exit network 1 // 6 G3|6 // 232566	Amn1	3.65E -03	1.32	3.62 E-03	1.32
172 131 79	ENSMUST00000027193 // Ndufb3 // NADH dehydrogenase (ubiquinone) 1 beta subcomplex 3 //	Ndufb 3	3.65E -03	1.47	2.53 E-02	1.33

172 066 85	---		3.65E -03	-1.24	1.02 E-02	-1.21
175 205 92	---		3.66E -03	-1.14	4.45 E-02	-1.09
173 122 93	NM_178646 // Tigd5 // tigger transposable element derived 5 // 15 D3|15 // 105734 /// B	Tigd5	3.67E -03	-1.14	3.34 E-04	-1.18
172 135 93	NR_028558 // Snora41 // small nucleolar RNA, H/ACA box 41 // 1|1 // 100217464	Snora 41	3.67E -03	-1.45	2.41 E-02	-1.32
173 282 01	NM_146066 // Gspt1 // G1 to S phase transition 1 // 16 A1|16 3.8 cM // 14852 /// NM_001	Gspt1	3.68E -03	1.16	3.20 E-02	1.11
175 341 57	ENSMUST00000046433 // Rnf113a1 // ring finger protein 113A1 // X A3.3|X // 69942 /// NM	Rnf11 3a1	3.68E -03	-1.17	2.36 E-02	-1.13
175 497 70	---		3.69E -03	-1.58	6.68 E-03	-1.52
175 493 28	---		3.69E -03	-1.29	1.68 E-03	-1.32
175 369 31	---		3.70E -03	-1.56	3.01 E-02	
